# Constructing living buildings: a review of relevant technologies for a novel application of biohybrid robotics

**DOI:** 10.1098/rsif.2019.0238

**Published:** 2019-07-31

**Authors:** Mary Katherine Heinrich, Sebastian von Mammen, Daniel Nicolas Hofstadler, Mostafa Wahby, Payam Zahadat, Tomasz Skrzypczak, Mohammad Divband Soorati, Rafał Krela, Wojciech Kwiatkowski, Thomas Schmickl, Phil Ayres, Kasper Stoy, Heiko Hamann

**Affiliations:** 1 Institute of Computer Engineering, University of Lübeck, Lübeck, Germany; 2 School of Architecture, Centre for IT and Architecture, Royal Danish Academy, Copenhagen, Denmark; 3 Human–Computer Interaction, Julius Maximilian University of Würzburg, Würzburg, Germany; 4 Institute of Biology, Artificial Life Lab, University of Graz, Graz, Austria; 5 Department of Molecular and Cellular Biology, Adam Mickiewicz University, Poznan, Poland; 6 Department of Computer Science, IT University of Copenhagen, Kobenhavn, Denmark

**Keywords:** biohybrid, self-organization, construction, biobot, robotics, hybrid

## Abstract

Biohybrid robotics takes an engineering approach to the expansion and exploitation of biological behaviours for application to automated tasks. Here, we identify the construction of living buildings and infrastructure as a high-potential application domain for biohybrid robotics, and review technological advances relevant to its future development. Construction, civil infrastructure maintenance and building occupancy in the last decades have comprised a major portion of economic production, energy consumption and carbon emissions. Integrating biological organisms into automated construction tasks and permanent building components therefore has high potential for impact. Live materials can provide several advantages over standard synthetic construction materials, including self-repair of damage, increase rather than degradation of structural performance over time, resilience to corrosive environments, support of biodiversity, and mitigation of urban heat islands. Here, we review relevant technologies, which are currently disparate. They span robotics, self-organizing systems, artificial life, construction automation, structural engineering, architecture, bioengineering, biomaterials, and molecular and cellular biology. In these disciplines, developments relevant to biohybrid construction and living buildings are in the early stages, and typically are not exchanged between disciplines. We, therefore, consider this review useful to the future development of biohybrid engineering for this highly interdisciplinary application.

## 1. Introduction

Biohybrid robotic construction, a potentially broad field, couples interrelated engineered systems and biological systems. In the related fields of bioinspiration and biomimetics, extensive approaches exist for a range of applications, including building design, materials, construction and robotics (see [1–4]). However, in this review, we look to biohybrid robotics not as a form of bioinspiration, but as a subset of robotic *hybrid societies* (see [5]), in which biological organisms and robotic elements perform collective behaviours in a self-organizing way. With this understanding, we can define biohybrid living buildings as those where robotic, mechanical and live biological elements—potentially also with user interaction—collectively accomplish built structures for human occupancy.

Construction is a relevant application for biohybrid robotics, as biological organisms excel at producing material with limited resources, and robots excel at flexible and programmable control. Though automation in architecture, engineering and construction (AEC) sectors is rapidly growing in popularity and sophistication [6], investigation of biohybrid robotics in this context is currently rare and is an emerging research trend. We are aware of two projects pursuing foundational research for biohybrid living buildings, one being our own *flora robotica*, for shaping biohybrid structures [7,8], the other being *Living Architecture (LIAR)*, for programmable energy and resource infrastructure in building components [9]. In this review, we do not address all potential aspects of biohybrid living buildings, but focus specifically on the process of construction, including operations like material deposition and shaping. For buildings where living organisms are involved in construction, we identify the essential challenge to be steering biological growth or deposition into shapes or patterns that perform building functions. These can include not only the structural system (perhaps of multi-storey height) but also building envelope functions such as shading, thermal insulation, moisture barrier, air barrier and delivery of building utilities. Though bio-mechanical hybrid structures can conceivably be constructed by manual manipulation alone, the growth times are likely to be long and the construction tasks laborious, suggesting the usefulness of automation. Furthermore, the inclusion of self-organizing robotic partners enables continual management of the full biological deposition or growth process, which inherently involves some degree of unpredictability. In order to guide and shape biological elements during construction, robots might indirectly influence the organisms through the construction and manipulation of mechanical scaffolds, or directly influence them by providing stimuli specific to the species.

As biohybrid construction has been infrequently studied so far, we review the approaches that could be foundational for future developments. Broadly, we first review robots that interact with biological organisms, then construction involving biological organisms, and finally construction involving robot collectives. We seek to answer the following broad questions, in a sufficiently concrete way to facilitate future study: (1) which biological organisms are known to responsively deposit, generate, or shape living or non-living material and what natural mechanisms are understood to modulate these behaviours? (2) What existing autonomous technologies interact, or could be expected to interact, with organisms and behaviours that fall into the aforementioned category? (3) What methods have been, or could be, used to incorporate living organisms or their depositions into construction outcomes or processes? (4) Which existing robot control, hardware and user-interface approaches are relevant to the management of construction processes that incorporate living organisms?

## 2. Hybridizing robots and biology

**Figure 1.**
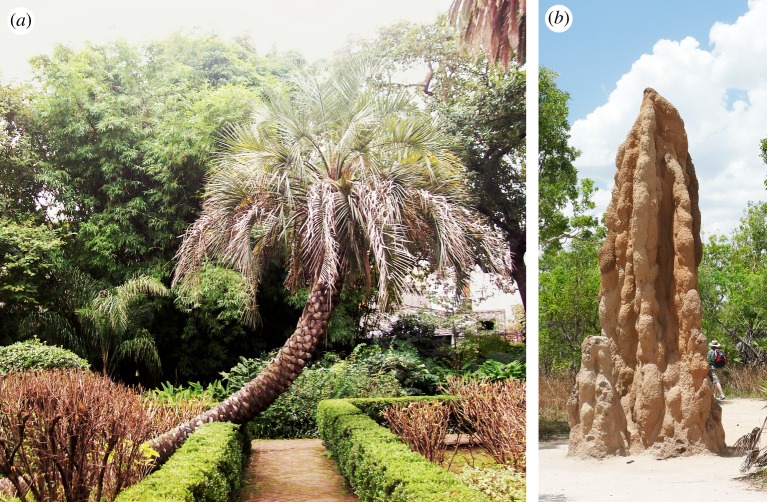
Natural methods of shaping and material deposition, found in plants and social insects. (*a*) A tree-shaped substantially by natural tropisms; image used with license. (Image retrieved from Wikimedia Commons, from username Roberto Fiadone. Used with Creative Commons license CC BY 3.0. Image copyright holder chose and approved the license at upload.) (*b*) A termite mound built with natural stigmergy; image used with license. (Image retrieved from Wikimedia Commons, from username Thomas Fuhrmann. Used with Creative Commons license CC BY 4.0. Image copyright holder chose and approved the license at upload.) (Online version in colour.)

Though studies investigating the construction potential of biohybrid robots are rare, many existing examples of robotic interaction with organisms could be foundational for novel applications. Plants and material-depositing animals are two major categories of organisms that are candidates for biohybrid construction (figure 1). In this section, we first review the behaviours of these two organism categories that could be useful for steering or shaping their deposition or growth into constructed artefacts. We then review robots that interact with biological organisms on various scales, including organisms that might not be directly applicable to the task of construction, as their approaches to interaction could be extended in useful ways.

### 2.1. Organisms that are candidates for biohybrid construction

#### 2.1.1. Material-depositing behaviours of animals

Social insects (e.g. ants, honeybees, wasps, termites), collectively construct ‘houses’ (nests) in a decentralized and self-organized way. Their construction occurs through low-level interactions among themselves and with their environment, which they continually reconstruct by building (general: [10]; ants: [11]; honeybees: [12]; wasps: [13,14]; termites: [15,16]).

Some simple mechanisms impact the insects’ patterns of material deposition or further shaping, such as thermoregulation [17,18], tunnel digging [19,20] or vibrational communication [21]. More complex mechanisms involve spreading of chemical gradients and modulation of animals’ behaviours based on the local concentration of these substances [22]. Such substances can be pheromones emitted by the queen, by the brood, or by building workers [23]. Alternatively to pheromone gradients, there can also be gradients in the density of the physical presence of brood, workers, or building materials, which can also function as a form-giving template [24]. Construction can be complexified by cascades of environment-changing behaviours that are triggered through environmental cues and signals—a phenomenon known as *stigmergy* [25]. To roughly summarize, stigmergy is a category of mechanisms by which social insects communicate among themselves not directly but by responding to the conditions found in the environment, which may have been modified by any of the insects [26]. One example of this is termite nest-building as shown in figure 1*b*, where the termites do not directly communicate about what to build, but rather simply respond to the already placed material in making their individual decision about where to place the next [25]. Another example is in how ants forage for food, wherein they again do not communicate directly, but rather choose their path based on the pheromone trails collectively left by the colony [27]. The presence of these behavioural feedback loops, and the nonlinearity of stimulus–response relationships, can lead to a significant increase in the complexity of the produced nests [10].

Beyond social insects, many animals construct their nests through material collection and deposition, including birds [28], badgers [29], mole rats [30] and beavers [31]. Beavers, as a prominent example, exhibit a construction activity that can be seen from a stigmergic perspective. The beaver not only constructs its nest by depositing material collected in the surrounding environment but uses this material to construct water dams which in turn heavily shape that environment. The resultant environmental changes can then trigger further building activities in the nest or dam (e.g. correctional restructuring depending on water level and water flow). Some animals also construct nests by depositing material they have secreted. Prominently, silkworms build cocoons from secreted protein forming strong fibres [32], somewhat similar to spiders weaving their nets [33].

#### 2.1.2. Modelling material-deposition by animals

The nest construction of paper wasps and termites has been modelled several times with qualitatively different approaches. For example, [34] intensively examines the search-space of ‘stigmergic rule scripts’ implemented in a lattice swarm model, finding several rule-sets that produce a paper wasp-like nest. However, the cognitive abilities of individual modelled wasps need to be strong in this approach, able to process 211 different nest configuration properties. Other studies show that an alternative approach—simple sets of a few locally applied rules—can also be derived from observing the wasps. These sets are capable of modelling the dynamics of nest growth, suggesting that the wasps may govern their construction behaviours using only a few simple rules based on simple local assessments [13,14]. As a construction principle, this looks rather general and applicable across many domains. However, the study of [35] suggests that behaviours evolved in nature are evolved for a specific animal, task and environment, and therefore the derived construction principle may not be useful for understanding animal construction generally.

In the related fields of bioinspiration and biomimetics, if the desired application closely resembles the conditions of the biological inspiration source, models have been successfully translated across physical spatio-temporal domains. For example, collective transport of material observed in ants has successfully been used as a modelling inspiration to develop control for autonomous robot swarms which collectively transport objects [36,37]. This suggests that extending such models to biohybrid cases, where robots and organisms collaborate, could be investigated. Modelling approaches for self-organizing robots are discussed further in §§ 4.2 and 4.3.3.

#### 2.1.3. Motion and tropism behaviours of plants

In addition to the behaviours of material-depositing animals, we look at the behaviours of plants that may be relevant for shaping biohybrid artefacts. Perhaps contradicting common perception, plants show a remarkable diversity of movements. Apart from passive propagules (detached pieces riding external forces) and motion due to purely physical processes (e.g. hydro-responsive curling in the resurrection plant [38]), there is a plentitude of physiologically controlled *active* growth and motion responses. *Active* plant movements can be grouped into:

(i) autonomous, endogenously controlled movements;
(ii) externally triggered non-directional responses (i.e. *nastic* movements), where stimulus location is irrelevant for response; and
(iii) externally triggered directional responses (i.e. *tropisms*), where stimulus location determines the direction of growth and motion, see example in figure 1*a*.

Of the autonomous movements, the most universal is circumnutation, which occurs in elongating tissues of all plants. This behaviour, whereby tissues wind around their mean growth direction, is most notable in climbing plants that wind around a support, such as the common bean or morning glory [39–41]. This basic motion interacts with other motion behaviours, especially irreversible tropisms involving growth. *Nastic* movements are typically fast and reversible responses where direction is incidental, such as the closing of a venus’s fly trap regardless of the excitement direction [42]. Because of the context of applying robot–organism interaction to construction, we focus on the directional *tropisms* of plants, reviewed below. In natural settings, many of these responses occur simultaneously, with the strength of each response weighted differently according to species, developmental stage, tissue and situation.

*Tropisms* are directed growth responses guided by stimuli and enacted through the plant hormone auxin. Plants react to a variety of environmental cues with tropic movements, particularly at the roots [43–45]. Tropic changes in growth direction occur by redistributing concentrations of auxin, triggering anisotropic growth and thus inducing curvature. Plants employ gravity as a primary spatial cue to orient their growth, via *gravitropism*. Stems generally grow against the gravity vector, while roots grow along it. Lateral roots, branches, or leaves often keep the gravity vector at a constant angle to their growth direction. Gravity is sensed in regions near growth tips (of shoots or roots) via subcellular statoliths [46], ultimately leading to anisotropic expansion and division of cells, causing directional re-orientation [47]. Even small gravitational forces (as little as 0.1 g) can produce profound effects on growth patterns (cf. wheat seedlings, [48]).

Plants react and adapt to mechanical impacts on all scales [49–51], from stretch-activated ion-channels in cell membranes to wind-swept trees minimizing surface of exposure [52,53]. Although gravity is a type of mechanical stimulus, the sensing and signalling pathways for gravitropic responses only partially overlap with those for other mechanical impact responses [54]. In general, mechanical forces provide plants with information about their environments and themselves, allowing for adaptive behaviour [55]. *Thigmotropism* (touch-guided growth) can readily be observed in root tips growing along the edge of dense soil clumps, assessing and following the penetrability of the material while still generally satisfying their gravitropism [56,57]. Another thigmotropic mechanism, common in climbing plants, helps tendrils coil quickly around objects they touch using ionic signalling and differential turgor-changes. If the stimulus is only transient, tendrils can uncoil again. However, if irreversible responses (growth and lignification) have already occurred, the coiling can no longer be undone [58,59].

Plants perceive light wavelengths from UV-B to far-red (280–750 nm), incorporating it in a number of ways. For example, the incident direction and duration of photoreceptor exposure is used to help time key developmental decisions and to continuously direct growth to exploit the most promising local light situation [60–62]. Additionally, light in the visual spectrum (400–700 nm) is a necessary food staple of plants and is absorbed via photosynthesis [63–65]. Concurrently, phototropism directs growth trajectories relative to the incident angle of light, for which the typical sensing mechanism is well-characterized. Blue light (and to a lesser extent UV light) excites membrane-bound proteins, relaying the signal to the cell or to responding tissues further away. This again leads to the same redistribution of auxin concentrations, and subsequently anisotropic growth [66–68]. Phototropic responses and their intensities vary largely across species, developmental stages, and tissues. For instance, some climbing plants will temporarily employ *skototropism* (growth towards shade) to find a support to climb, by growing towards the darkest spot, but not necessarily away from the brightest. There are also reversible directional responses to light, such as the light-stimulated movement of leaves [69,70] or the famous heliotropic movement of young sunflowers before the flower opens [71].

Being photosynthetic organisms, actively avoiding shade is a major benefit to plants. They have evolved complex strategies to manage shade or potential shade by harnessing their full arsenal of light receptors [72]. These strategies include the avoidance of projected future shade from nearby competitors by triggering the well-researched shade avoidance syndrome (SAS) [73]. This response is triggered by spectra enriched in far-red (and possibly green: [74]) light, a good indicator of the proximity of chlorophyll-bearing organisms. Mechanical stimulation and plant-emitted volatile chemicals can also feed into this response [61,73]. It usually results in elongated stems and in petioles with reduced branching and root growth. Meanwhile leaves tilt upwards (*hyponasty*) in an attempt to outgrow competitors. Much less is known about shade-tolerance mode, which is employed by plants growing under a dense canopy to cope with long-term shaded conditions. Typically, this response leads to an increase in specific leaf area (SLA), an optimization of photosynthesis for low-light conditions, and greater physical defence of leaves [75].

*Chemotropism* (chemically guided growth) has long been known in roots, which sense a plentitude of chemicals and are seemingly aware of local and global needs [76]. In shoot tissues, chemotropic growth has been shown in the parasitic dodder, as it seeks and selects host plants in a dark environment [77,78].

Plants control which tissues follow which environmental cues, as well as the timing and magnitude of response. In this way, a certain stimulus can influence or fully override the direction growth would otherwise follow, according to factors like nutritional status [44]. The development of a climbing bean is an illustrative example of this concept. First, the germinating bean shoot grows against gravity, but towards (blue) light. Soon, autonomous circumnutational winding sets in, allowing the plant to use its sensing machinery to assess the environment in much higher spatial resolution [79], while increasing the odds of hitting and encircling a support. If that occurs, thigmotropic cues help the bean wind around the structure, while the other tropisms are still present. More favourable light regimes allow the bean to climb supports at more horizontal slopes, while both light and gravity positively influence the circumnutation radius. Finding a support triggers a change in development as the plant is relieved of the need to mechanically support itself [39,58,78]

All of these processes and sensing strategies are at the disposal not only of herbaceous species like bean, but of self-supporting woody species. Such species have been used in the domains of architecture and plant shaping (see §3.3) to build up adaptive living support structures over years or decades. The guidance of woody species through the stimuli and tropisms described here, rather than through manual manipulation, could be investigated. Beyond using the plants’ natural growth and motion behaviours, the genetics of plant development are increasingly becoming understood [80], opening routes to ‘programming’ plants for functional applications like construction.

#### 2.1.4. Modelling plant growth and motion

A generic formalization that models the comprehensive biological phenomena of plant growth and motion across species does not yet exist [81,82], but the many approaches described in the literature are extensive, diverse and sophisticated. Many models have been proposed (cf. reviews in [53,83–87]), ranging from abstract geometric models to detailed biological models of the motion behaviours described in §2.1.3. Overall, we can roughly group the examples in the literature into the categories of (1) abstract models or grammars inspired by plants, (2) computer graphics models for plant visualization and (3) biological models of observed plant dynamics. Though the topic of plant modelling is too broad for us to comprehensively describe, in this subsection we review some highlights from these categories, focusing on relevance to biohybrid robots.

Arguably the most prominent type of abstract model or grammar inspired by plant development is L-systems [88–90]. An L-system is a formal language with a parallel rewriting mechanism where a set of context-free generative rules are applied to a set of symbols starting from an initial seed. Many variations of L-systems are described in the literature, mostly with the purpose of extending the system to react to environmental factors during development. In the approach of [91], the symbols of the L-system are agents of different types and their interactions and dynamics are defined by a swarm grammar. Others have introduced the concept of virtual plants explaining the development of plants interacting with the physical and biotic environment [92]. Some of the other methods of modelling an individual plant’s morphogenesis are proposed by Bell [93] and Niklas [94], complemented by the approach of [95] for a plant’s motion. Some models are introduced to capture other aspects of growth in plants. For example, the approach of [96,97] uses a swarm intelligence approach to model morphogenesis, inspired by plant resource distribution in response to environmental factors. Many of these models could be investigated for the control of self-organizing robots in a biohybrid system, particularly in combination with approaches discussed in §§ 4.2 and 4.3.3. They might also be extended for integration with biological plant modelling data, as [98] explore by integrating L-systems with multi-scale tree graph (MTG) data structures, a common multi-scale representation of plant architecture in biological sciences.

Modelling plants is an expansive and relevant topic in computer graphics and animation. One general approach uses generative models, such as the abstract models and grammars described above, to simulate shape and development of plants, reaching desired shapes by tuning parameters (e.g. [99–101]). Another approach takes a hand-drawn sketch or an image of a plant as generative input and uses it to construct a visually realistic three-dimensional (3D) model. In sketch-based modelling (e.g. [102,103]), a user draws a sketch of the plant and the system approximates parameters of a base model in order to construct the 3D plant shape. In a similar approach (e.g. [104]), sketch gestures from the user interact with the plant model to steer and shape it with simple brushstrokes. These sketch approaches can be combined with self-organizing models (e.g. [105]) and could be investigated in the context of the human–biohybrid interfaces discussed in §4.4. In image-based modelling, images of real plants are processed by methods of computer vision and image processing, and an optimization method infers the parameters for a graphical model of the plants. For example, [106] use a differential evolution method to retrieve a plant model from the real images taken from the trees, incorporating its growth, sway in the wind, and addition of leaves. A similar method on the forest scale is reported by Zamuda & Brest [107], while [108] present a different extension using a laser scan rather than image. Though these approaches currently focus on computer graphics, they could potentially be investigated for extension to data-driven models of plant response to stimuli in biohybrid set-ups, similar to the simple approach of [109] described below.

Biological models are relevant to the application of biohybrid robots, especially if they can be used to predict or simulate a plant’s response to specific robotic stimuli. We are not aware of any existing models that can universally fulfil this need when engineering biohybrid systems with plants. *Ad hoc* approaches to this problem (e.g. [109,110]) construct a data-driven model by image processing time-lapse records of a certain species in a given set-up, from a few initial experiments. More generalized approaches could be investigated, building from a variety of models in plant science literature. Though many approaches exist for agricultural purposes to improve crop yields (see example review by Malézieux *et al.* [111]), these are not likely to extend to the application of construction. Other plant science approaches, however, focus on the growth patterns, trajectories and biomechanics of individual plants, and are therefore adjacent to the engineering task of steering and shaping growth through automated robotic stimuli for biohybrid construction. Arguably the most relevant for this engineering application are unified models of several tropisms (see §2.1.3 for description of tropisms) such as that presented by Bastien *et al.* [112], or comprehensive models of growth in a specific species (e.g. [113]). Other relevant approaches focus on a variety of topics, including generalized measurement of growth volume [114]; image processing for spatio-temporal leaf and root patterns [115]; genetic impacts on growth trajectories [116]; impact of photosynthesis patterns on growth’s response to resources [117]; geometry of nutation and its relation to growth dynamics [41]; and building a framework for simulation of growth and development [118].

### 2.2. Robots that interact with organisms

One approach to biohybrid robotics described in the literature is to use engineered tissues as part of the machine [119,120]. In this review focused on construction as application, we review robots that influence intact organisms, as we are interested in their behaviours of depositing or growing building material. Robotics that incorporate biological organisms can have any of the following interaction types:

(i) microscale (i.e. coupling with individuals),
(ii) mesoscale (i.e. interaction with groups, as artificial agents or via local stimuli), or
(iii) macroscale (i.e. globally influencing environment).

Of the below robots interacting with animals, not all are with organisms that are useful for construction. However, their approaches to interaction could be investigated for animals with material-depositing behaviours.

#### 2.2.1. Coupling with individual animals

Today’s technology fails in delivering centimetre scale robots which are able to perform autonomously and effectively in unknown dynamic environments. In contrast, natural insects are able to easily navigate in most environments while successfully maintaining control and stability. Therefore, as a compromise, a biobiotic approach (i.e. cyborg system [121]) could be followed, allowing the wireless control and navigation of insects to perform meaningful tasks in such environments. For example, cockroaches with backpack systems are manoeuvred wirelessly to perform line following behaviour using neural stimulation [122], and augmented rats could be guided by visual cues and solve mazes [123,124]. The ZigBee enabled backpack system is equipped with a tissue-electrode bioelectrical coupling system which insures safe electrochemical stimulation. Erickson *et al.* [125] further investigate the locomotion response to various degrees of neuro-electric stimulation on the Madagascar hissing cockroach (*Gromphadorhina portentosa*). Investigation has also been done for bio-machines (i.e. mechanical cyborgs), where sensing or actuating in a robot is accomplished in part by biological tissues; [126] have shown robot propulsion with frog muscle tissue.

#### 2.2.2. Interaction with groups of animals or environments

Animal behaviour as a response to events in the environment or to local interaction between group members has been modelled by several methods, described above. Robotics approaches can allow further investigation of animal behaviour, by replacing swarm individuals with biomimetic robots and then establishing cause-and-effect interaction sequences. The ASSISI Project (Animal and robot Societies Self-organize and Integrate by Social Interaction) [127], introduced a biohybrid society composed of animals (e.g. fish and honeybees) and robots. First, the robots interact with the animals, learning their behaviour and adapting to it in order to be socially accepted. Then, they feed information into the society through physical channels, influencing the system to move towards desired states. Robots and animals can make collective choices in their habitats, while the robots couple separated habitats by sharing information between them [128].

In one approach, [129] develop autonomous robots integrated into groups of live cockroaches to influence collective decision-making. The robots were designed to exhibit similar behaviour to cockroaches and were coated with a chemical blend to bear an acceptable chemical signal. In this work, the robots were able to introduce bias into the decision-making process by influencing the cockroaches into aggregating towards a less favourable shelter. da Silva Guerra *et al.* [130] follow a different approach for physical acceptance within living crickets (*Gryllus bimaculatus*). By installing decoys (live cricket heads) on the robots to increase the acceptance and allow for proper interaction, the robotic crickets were able to trigger specific insect behaviours by performing certain repeated movements (e.g. courtship or agonistic behaviour). Also, in the Chicken Robot project [131], a mobile robot (i.e. PoulBot) was developed to collaborate and control a group of chicks. Based on a learned filial imprinting model, the robot was able to integrate and show leadership behaviour using acceptable movement patterns and appropriate emitted sounds.

**Figure 2.**
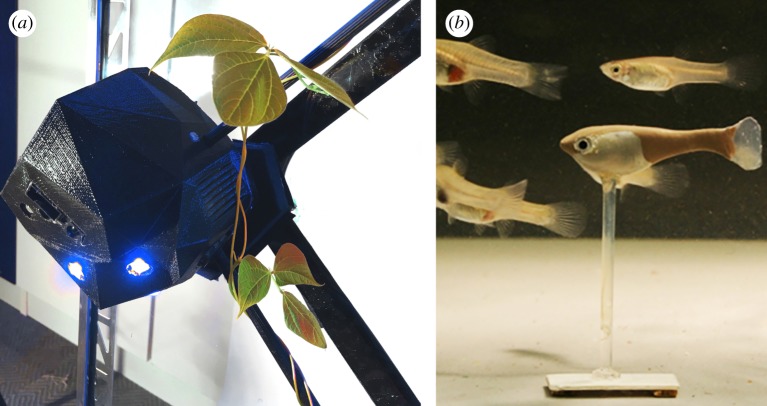
Two approaches to interaction between robots and natural organisms. (*a*) A robot interacts with plants by providing directional light stimuli, as seen in [134,135]. Image by authors. (*b*) A robotic fish interacts with a group of natural fish as an artificial agent in [136]; image from [136] and used with license. (Image reprinted from fig. 1*d* of the Royal Society Open Science paper of Bierbach *et al.* [136], DOI, open access. Used with Creative Commons license CC BY 4.0. Authors holding the image copyright approved the license at publishing.) (Online version in colour.)

To investigate interaction with marine animals, [132] construct a robotic fish (stickleback *Gasterosteus aculeatus L.* replica) which can be remotely controlled to move around in a fish tank. The robotic fish was able to exhibit leadership behaviour by recruiting a single fish from a refuge, and by initiating a turn in singletons and in groups of 10. An interesting observation is that the individuals would respond to the robotic fish to a greater degree than to others. The reasons for this could be the behavioural model (i.e. the robotic fish moves faster than other fish and without stopping) or positioning (i.e. the presence of the robotic fish at the front of the group). In similar work [133], see figure 2*b*, experiments were conducted implementing the following behavioural patterns with guppies (*Poecilia reticulata*): swarm following, integration, predator, and recruitment behaviours. Interestingly, a robotic fish was able to recruit a group of fish to the non-favourable area at the centre of the tank. Executing a sequence of behaviours (first integration then recruitment) helped the robotic fish to be integrated and accepted within the swarm, hence, succeeding in its recruitment mission to the desired target points. Later, [136,137] investigate acceptance of the robotic fish within the swarm in further detail. The results indicated that natural appearance and motion significantly increases the acceptance level of the artificial individual. Hence, the precise modelling of animal behaviour and individual characteristics is crucial. Along this line of work, [138] develop a robotic fish (zebrafish *Danio rerio* replica) which can beat its tail with different frequencies and amplitudes. The experiments concluded that the tail beating rate increases the acceptance level of the robotic fish within the shoal.

The safety of both animals and robots is important within biohybrid environments. The classical robotic task of collision avoidance was re-approached by Gribovskiy & Mondada [139] and Gribovskiy *et al.* [140] using methods such as fuzzy control with the constraints of the new systems. Interesting tasks for this system are mapping and exploration [141] where the topological information about an unknown environment is obtained based on local interactions without localization. Whitmire *et al.* [142] follow an acoustic approach where the biobots are equipped with a microphone. The swarm of biobots was able to localize a sound source which allows further investigation in search and rescue applications. In a similar context of search and rescue missions, the concept of an invisible fence composed of biobots as a reliable wireless sensor network is introduced by Latif *et al.* [143]. Also, the approach allows the biobots to guide each other towards light sources in order to charge their batteries using solar energy in extended mission durations. Yang *et al.* [144] introduced a protocol for manoeuvring spiders. The spiders were steered successfully in the left or right directions using electrical simulation. This work is considered an important step towards creating a spider biorobot.

Research has also dealt with technological intervention at the scale of full ecosystems, via distributed sensing, tracking, and monitoring of wildlife [145], including animals that can exhibit self-organizing behaviours in groups, such as birds [146,147]. Beyond monitoring, the restoration of overall ecosystem health via mobile robotics has been proposed, to increase biodiversity and combat desertification [148].

#### 2.2.3. Coupling with individual plants

Robot actuators have long been developed to handle or harvest individual plants or organs in greenhouse settings, cf. [149,150]. Recent developments trend towards deeper integration. Technological coupling with plants to form biomachines (i.e. botanical cyborgs) has been explored for sensing, display, and actuating [151]. One way of interacting with plants is via their chemical and electrical signals [152,153], which perform even long-distance communication [154]. Robotic effects on plant signalling are used in plant science research to understand physiological behaviours [155]. Physiological responses of the plant to the environment have been suggested as a basis for bio-sensors or phytosensing (where the environment can be sensed indirectly via the plant). The PLEASED project uses plant roots as an organic approach to a distributed sensor network [156], while a plant and mobile robot pair [157], and the *flora robotica* project, use the plant as a sensor to inform devices [8]. Plants as bio-sensors has become a developed research topic for environmental monitoring [158], and engineered plants have been proposed even for especially challenging environments [159]. Plants have also been used to power very low-voltage devices [160]. By infusing organic conductive polymer into a cut plant’s vascular system, a plant has even been used as functional circuitry [161].

Research on steering the morphological development of individual plants is rare, as agricultural concerns, for instance, do not motivate such studies. However, there is a line of research on shaping plants that develops an automated process of evolving controllers that direct the growth of a single plant to certain goals [109,110,162,163]. Machine vision was used to understand the behaviour of single bean plants in reaction to external light stimuli, and to construct data-driven models of the plant’s growth and motion. The models were used to control light stimuli and steer the plants to predetermined targets, adaptive targets, and around obstacles—in simulation and on real plants. This approach is extended to robots with distributed control, providing stimuli to guide the decisions of climbing plants, between several growth path options [134,135], see figure 2*a*. Similar methods applied on a much larger scale could drive more complex construction processes with plants.

#### 2.2.4. Interaction with groups of plants or environments

A plant-inspired robot has been developed in the Plantoid project to mimic a root system [164], in research towards soil monitoring. As root systems of plants use forms of indirect communication, similar plant-inspired robots could feasibly integrate into a group of real plants to influence their behaviours, similar to approaches for robot interaction with social insects described above. Automated vehicles and robots are commonly used for industrialized agriculture, automated greenhouses, and home gardening (e.g. [165–172]) for an expansive range of tasks (see [173]) due to their precision or cost efficiency in monitoring and supporting plant growth [174,175]. Automation approaches have been developed even for especially challenging tasks like weed control [176]. Guidelines have also been introduced for the design of plant nursing robots [177]. Computer vision and other imaging techniques for monitoring and 3D modelling of plants are also well-developed [178–180]. Steering of plant behaviours is again less explored. However, groups of plants steered by stimuli have been proposed as interactive displays for user devices [181,182].

## 3. Hybridizing buildings and biology

The majority of existing biohybrid construction uses some combination of biological organisms, manual manipulation, and static scaffolds or moulds. These generally hybridize biological and mechanical elements, without incorporating automation. Examples that include robotic elements are limited, and usually focus on autonomously maintaining organism health, rather than steering motion or shaping morphology. Current bio-mechanical hybrid structures can be roughly organized into the following categories:

(i) static mechanical scaffolds that support biological organisms;
(ii) biological energy sources in buildings;
(iii) plant growth shaped into load-bearing elements; and
(iv) forming building components from amorphous living material.

When shaping material into a fully equipped long-term occupancy building, the roles to be materially performed include not only the structural system but crucial building envelope functions (e.g. thermal insulation, moisture barrier, utility delivery). In this section, we review examples where a biological element fulfils one or more of these roles. Because infrastructural roles such as light emittance are often coupled to a material role such as utility delivery, the distinction is not always clear. Therefore, the works here include some examples that, though primarily infrastructural, we consider to be integrated into material building components in a way that might impact artefact shaping.

On one hand, in examples of static structural scaffolds hosting organisms or of building components that cultivate energy sources, the grown or deposited biological material typically does not carry the primary structural load, but rather contributes to a building envelope role. On the other hand, in examples of plants shaped into structural elements or of amorphous material shaped by moulding, the biological material often acts as the primary structural system, with envelope roles sometimes fulfilled either by artificial elements or biological ones. To realize biohybrid living buildings, the approaches described below could be individually extended, or potentially could be combined together in a variety of ways, such that plants, material-depositing animals, and microorganisms might coexist in a single living building. This section concludes by identifying opportunities in the reviewed bio-mechanical systems for extensions that integrate robots as partners in shaping biohybrid artefacts.

### 3.1. Static structural scaffolds that host biological organisms

Structural scaffolds that incorporate organisms are organized such that artificial elements form a mechanical scaffold upon which the biological elements can grow or deposit material. The mechanical scaffolds are static, steering biological growth or deposition through their predetermined shape and arrangement of components. The scaffolds leave voids for the biological elements to fill, or form paths or surfaces for them to follow. After biological material has been added, the mechanical scaffolds stay in place as a permanent part of the structure.

#### 3.1.1. Scaffolds for animals depositing material

**Figure 3.**
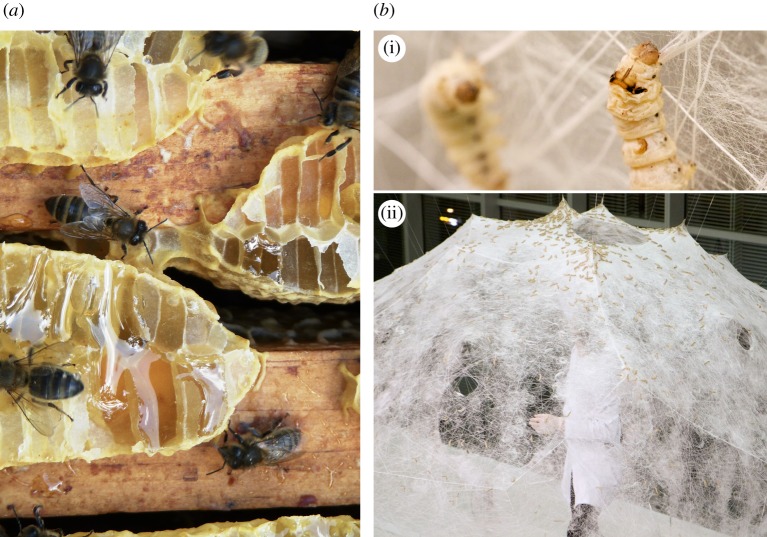
Natural material-depositing behaviours of animals in response to their environment. (*a*) Social insects such as bees will naturally build structures that are adaptive to their environment, for instance by filling gaps with honeycomb; image used with license. (Image retrieved from Wikimedia Commons, from username Onésime. Used with Creative Commons license CC BY-SA 3.0. Image copyright holder chose and approved the license at upload.) (*b*) Silkworms constructing a domed pavilion, by depositing material according to the shape of the mechanical scaffold in their artificial environment [183,184]. Top and bottom images both used with license. (Top image and bottom image both retrieved from Wikimedia Commons, both from username Sj. Both images used with Creative Commons license CC BY-SA 4.0. Image copyright holder for both images has approved the licenses, verified by OTRS ticket number 2016072510000875.) (Online version in colour.)

In structural systems involving animals that exhibit material-depositing behaviours, mechanical scaffolds are designed to steer deposition patterns specific to the species used. Silkworms are guided by density in the scaffold, while honeybees are guided by voids. In the *Silk Pavilion* project by Oxman *et al.* [183,184], shown in figure 3*b*, a domed room-sized scaffold forms the substrate for silkworms to deposit their threads. The scaffold comprises frame modules, each of which is prefabricated and robotically wound with a sparse pattern of silk threads. When released, the silkworms seek to patch gaps in the pattern of existing silk threads, as they naturally would for cocoon-building. The silkworms do not cover the entire scaffold in dense silk fibres—rather, their deposition is guided by density of the robotically wound threads, as they are not able to cross gaps larger than their body size. Therefore, intentional windows in the sparse pattern of the scaffold are maintained when the silkworms fill in their dense matte of fibres. In the *Co-occupied Boundaries* project by Ilgun & Ayres [185], an object-sized 3D printed polymer scaffold is shaped to leave voids for honeybees to construct their comb according to their natural behaviours, as shown in figure 3*a*. The printed polymer filament forming the scaffold is dense, but maintains gaps large enough for honeybees to pass through, giving them pathways to all sides of the scaffold. The rough material texture of the scaffold and the sloping angles of its sides create surfaces to which the honeybees can easily attach comb. The placement of comb is guided by creating large voids with two or more sides of enclosure. In both of these examples, the mechanical scaffold must be structurally sufficient to support the load of the biologically placed material. In the case of the [183,184] *Silk Pavilion*, the fibres placed by the silkworms are not self-supporting and cannot serve a structural role on their own. In the case of the [185] *Co-occupied Boundaries*, the honeybee comb is self-supporting once formed, although it requires a scaffold for initial placement. The structural properties of the comb are not further investigated by Ilgun & Ayres [185], but due to the wax material of comb, it is unlikely that it would be able to support large external loads.

Material deposited by animals, while often capable of serving some structural role on the scale of the associated animal, is unlikely to be stiff enough on its own to carry building scale loads or human occupants. Stiffening methods such as resin impregnation could be investigated for these materials to prepare them for a structural role, but this may be a prohibitively inefficient construction process. Alternatively, these materials could be investigated for non-structural roles in building construction, such as thermal insulation or façade cladding.

#### 3.1.2. Scaffolds for microorganisms

Microorganisms are integrated with mechanical scaffolds as part of structural systems, as well as for other functional roles such as the cleaning of pollution. For structural systems, [186] cultivate bacterially produced cellulose on 3D printed polymer scaffolds. The bacterial cellulose grows to fully coat the surfaces of the scaffold, and additionally forms membranes across gaps. Similar to the silk fibres described above, these cellulose membranes are unlikely to bear building scale loads, but might be investigated for other roles such as thermal insulation or moisture membranes. In a different approach, mycelium fungus is investigated for soil decontamination by Sollazzo *et al.* [187] in their *Symbiotic Associations* project. The mechanical scaffold, in this case, does not serve a structural role for a building, but exclusively supports the growth of the fungus. Though not a direct part of the typical construction process, this approach could be investigated for use on the larger building site or as part of a structure’s foundation.

#### 3.1.3. Scaffolds hosting plants or habitats

The combination of scaffolds and plants may be generally familiar through gardening practices, such as the use of a trellis to host a climbing plant. For buildings, basic mechanical scaffolds on façades and roofs have been used extensively in building construction to host plants as green walls and green roofs [188,189]. This strategy is exemplified in façades designed by Patrick Blanc, as described by Gandy [190]. The plants, and especially the soil mass required to host the plants, serve a substantial thermal insulation role and may also work to mitigate the urban heat island effect [191] and manage urban stormwater [192]. The full range and limitations of the economic and environmental aspects of green roofs and other green infrastructure are for instance examined in [193]. Examples in the literature work to advance the flexibility or functionality of green walls approaches. For instance [194] investigate 3D printed solutions for suitable growth substrates, achieving flexibility in geometry and in fabrication processes. Another approach to increase flexibility is taken in the *Plug-In Ecology* project by Joachim [195], where plants are individually hosted in modular building components that can discretely pop in and out of a larger structure. Besides flexibility, the functionality of plants on mechanical scaffolds is increased in the *Eco Boulevard in Vallecas*, by Ecosistema Urbano *et al.*^1,2^ and in the *Baubotanik Plane Tree Cube Nagold*, *Baubotanik Tower*, and *Baubotanik House of the Future* by Ludwig *et al.*^3,4^ and Ludwig & Schönle [196], in all of which trees are planted upon an open structural frame that is either temporary or permanent and are grown to fill in gaps and form the façade of the building or to form the load-bearing structure, rather than be added to an existing fully enclosed façade. Providing an alternative functionality, although not implemented in a building, the floating artificial islands in the [197] *RiverFIRST* project act as a simple scaffold like that of a green roof, to support a range of plants and animals present naturally in local habitats, with the aim of increasing biodiversity (cf. urban biodiversity, [198]). The systems described above, and similar, typically incorporate some robotic elements for automated irrigation, monitoring, and maintaining health of the plants. However, none of the aforementioned examples, or similar green walls we found in the literature, use their robotic elements to steer the location or shape of growth.

### 3.2. Biological energy sources in buildings

Cultivation of algae or microorganisms as energy sources in buildings is an approach that typically incorporates automation to manage the infrastructural system and keep the organisms healthy. Some examples are integrated into building components in a way that impacts envelope functions or artefact shaping.

#### 3.2.1. Growing algae for biomass

**Figure 4.**
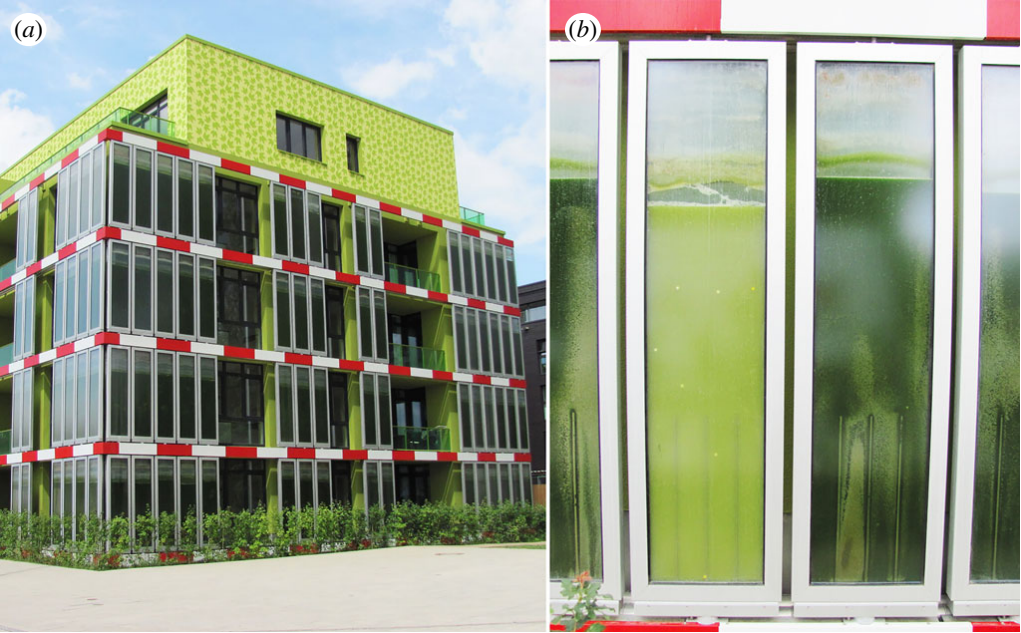
The *BIQ Algae House* [200] with algae façade panels by Elsayed *et al*. [201] that cultivate biomass for energy production. Left and right images both used with license. (Left image, titled ‘IBA Hamburg BIQ (2).nnw.jpg’, and right image, titled ‘IBA Hamburg BIQ Fassadenteil mit Mikroalgen.nnw.jpg’, are both retrieved from Wikimedia Commons, both from username NordNordWest. Both images used with Creative Commons license CC BY-SA 3.0. Image copyright holder for both images chose and approved the licenses at upload.) (Online version in colour.)

Algae are systematically cultivated and harvested for biomass in dedicated photo-bioreactor plants, as reviewed by Proksch [199]. Integrating this process into buildings allows the cultivation to occur on its site of eventual use, cutting down on transportation energy or on distribution losses. A fully operational example of integration can be seen in the [200] *BIQ Algae House* by architect Splitterwerk, shown in figure 4. The algae façade panels by Elsayed*et al.* [201] act as mobile shading devices for the building interior, in addition to their role of continual energy production. This approach of designing the integrated algae cultivation system to serve additional standard building functions is also explored by Decker *et al.* [202], in the relationship between algae density in the panel and interior light levels and distribution. Both of these examples use rigid façade panels that are made to be mounted in a specific way. Systems with greater flexibility in use case allow cultivation in interiors of buildings or as part of urban infrastructure. The *HORTUS* project by Pasquero & Poletto [203] cultivates algae indoors and incorporates user interaction as part of the CO_2_ and oxygen ventilation loop. The *Urban Algae Canopy Module*, as described by Ednie-Brown [204] prototypes algae cultivation modules for use in public plazas and other urban infrastructural spaces. The modules can provide an additional function of shading, similar to the façade panels described above, but do so in the form of a canopy over open outdoor space.

#### 3.2.2. Microorganisms as light sources

Bioluminescence has been investigated for infrastructural applications [205], including bio-lighting in cities and a few preliminary studies for bioluminescent building components. In the *Biolamp* project by Genetic Architectures Research Group & Estévez^5,6^ small discrete containers of bioluminescent bacteria are integrated into a domestic interior to test whether useful light levels can result. By including a high density of containers, a low but useful level of ambient green light was achieved, but keeping the bacteria healthy in such a decentralized organization was considered too challenging for the method to be pursued further [206]. The *Microbial Home biolight* by PHILIPS^7^ addresses this bacterial health challenge by consolidating larger containers in a single location, and connecting each container to a source of methane gas from an onsite biodigester [207]. In *Bioluminescent Field*, a spatial art installation by Burggraf *et al.*,^8^ instead of using bacteria with a constant glow, containers that can be manually agitated by users are filled with microorganisms that glow only when disturbed [208]. Providing robotic stimuli to trigger bioluminescence in buildings when desired, rather than uniformly, could be investigated.

### 3.3. Guiding plant growth into load-bearing elements

Many plant species do not require external support, and their property of providing material with low resource cost can easily be seen as advantageous for building construction. However, it is less automatically clear that plants can fulfil structural roles for occupant loads and multi-storey buildings. Existing examples of guiding or constraining plants into structures mostly have been made by handcraft practitioners or through indigenous traditions, partly because grown structures that are substantially large at present must have been begun years or decades ago. These approaches include manually rearranging roots, weaving stems, constraining stems into bundles, joining stems through grafting, and constraining stems onto temporary moulds. As a whole, these examples give evidence for the ability of plants to perform certain structural or building envelope roles. Newer studies in scientific or engineering fields extend these handcraft approaches, for example by embedding permanent mechanical elements into natural growth to perform supplementary roles (e.g. floor plates, handrails), or by using robotic elements to guide or shape plants through provision of stimuli.

#### 3.3.1. Manually guiding growth in the *Living Root Bridges*

**Figure 5.**
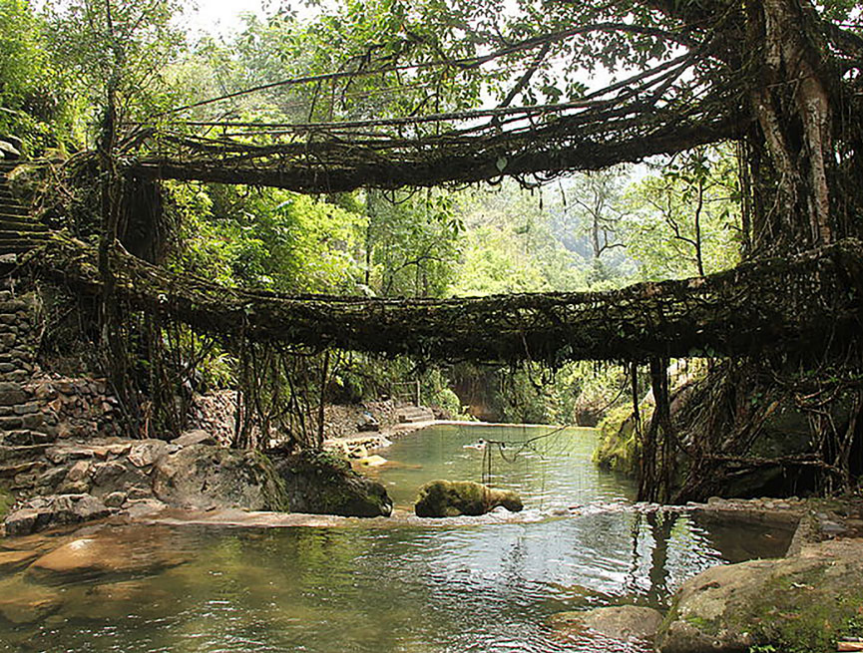
One of the *Living Root Bridges* constructed by the manual rearrangement of root growth over long periods of time [209]; image used with license. (Image retrieved from Wikimedia Commons, attributed to Arshiya Urveeja Bose. Used with Creative Commons license CC BY 2.0. Image copyright holder chose and approved the license at upload.) (Online version in colour.)

Several examples of building-sized structures, functioning successfully for occupant loads, can be seen in the constructions termed *Living Root Bridges* in Meghalaya, India (figure 5). As described by Shankar[209] and Chaudhuri *et al.* [210], these bridges, made from live plants over a period of years or decades, are demonstrated to structurally outlast steel suspension bridges in the area due to high levels of moisture and dynamic loads such as flash floods. According to [209], the *Living Root Bridges*, once constructed, can last for centuries with minimal maintenance, and are even used in the area to replace failing cable bridges. Shankar [209] documents the following process of light manual guidance of natural growth by which the bridges are formed over a period of 15–30 years: first, a hollowed tree trunk supported by bamboo scaffolding is used to guide young, pliable *Ficus elastica* roots across a desired bridge location, sometimes from both sides; second, multiple layers of ficus roots are guided through the trunk until the combined roots are self-supporting and the trunk is removed; third, multiple layers of roots are guided along the bamboo scaffold, until they too are self-supporting and the bamboo is gradually removed; finally (or simultaneously with the previous step), ‘dead load’ such as stones, wood planks and dirt are added to fill gaps and to test the bridge for structural stability. According to [209], mature bridges can carry loads of up to 35 people.

#### 3.3.2. Mechanically constraining growth

While in their young, pliable state, plant stems can be manually placed in a desired position, and then mechanically constrained in that position. Stable structures can, for instance, be built with pliable woody species such as willows, although the individual stems have low stiffness, by permanently constraining the stems in tightly woven patterns or in large, strong bundles. Over time, the individual plants sometimes graft with their constrained neighbours, but we are not aware of any examples where grafting is demonstrated to give additional load-bearing capacity to bundled stems. Examples of living willow construction are partially reviewed by Ludwig [211] and more generally reviewed by Gale [212] in their respective literature reviews. Gale [212] notes that the construction methods used for these living structures are based on ancient Sumerian techniques for building with cut reeds, currently still used in Iraq. Though these reed structures use dried plants rather than live plants, their methods of bending and constraining can be extended to live willows. Some of the simpler reed structures, described by Mandilawi [213], closely resemble many of the living willow structures. However, a significant category of reed structures—termed *mudhifs*—are more advanced, able to serve standard building functions for long-term occupancy. New *mudhifs*, according to [214], are currently underway that include water and electricity utilities, allowing functions such as cooling, refrigeration and internet connection. Though the *mudhifs*, historically documented by Broadbent [214] and analysed by Mandilawi [213], are made from cut and dried reeds, their construction techniques could be investigated for buildings made from living plants.

In the existing living willow structures, the woven or bundled stems form a structural frame, but not a fully enclosed interior. Two methods are documented in the literature for adding a façade or canopy to shelter occupants from wind or rain. One method, for tightly woven living willows, is to allow the foliage that grows from the stems to cover the small gaps in the woven structure, as seen in the *Living Willow Tunnel* by Gale.^9^ This does not provide a full enclosure, but can effectively buffer wind or rain if growth is allowed to mature for several weeks. The method can also be used for bundled structures, despite the much larger gaps, by following a longer construction process as seen in the *Hopland Willow Dome* by Schaeffer *et al.*^10^ In this application, as the willows in the bundled structure mature and grow branches, the new shoots are periodically constrained in locations where denser cover is desired, until the branches are thick enough that their foliage can buffer rainfall. A thick canopy was achieved in the *Hopland Willow Dome* within six years of growth, as documented by Calkins [215]. The second method is to use the living willows as structure only, and to use typical building materials to shade and shelter the structure’s interior, as seen in the tensioned textile roof of the *Rostock Willow Church* by Kalberer & Strukturen.^11,12^ In the built examples using these two methods, their respective canopies provide some degree of shelter, but they are far from full enclosure for long-term occupancy. By contrast, the *mudhifs* described above include fully functioning façades and roofs, with architectural details like columns, vaults, windows and doors (see [213,214]). The finished *mudhifs* use exclusively constrained reeds to form these architectural details, as the structures can be untied and reassembled on other sites, according to [214]. These *mudhif* construction techniques, so far used only for dried reeds, could be investigated to extend living willow structural frames into fully enclosed living willow buildings for long-term occupation, depending on whether the plants can be kept healthy in such a dense structure.

**Figure 6.**
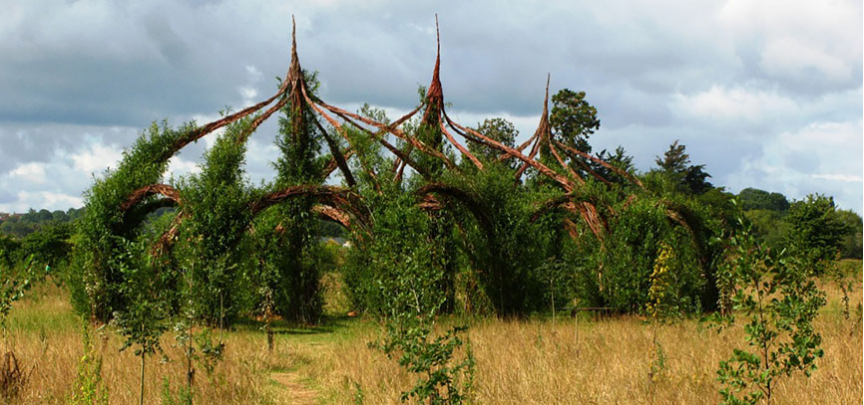
The *Longrun Meadow Willow Cathedral*,^15^ an example living willow structure, built by permanently constraining the willow in large bundles; image used with license. (Image retrieved from Wikimedia Commons, from username Geof Sheppard. Used with Creative Commons license CC BY-SA 3.0. Image copyright holder chose and approved the license at upload.) (Online version in colour.)

Weaving and constraining willow is popular for handcraft of living sculpture, furniture, and small building elements such as fences or garden tunnels [212,216,217]. Larger structures that exist in the literature are constructed by bundling willow rather than weaving it, and have been constructed from 1985 onward by Marcel Kalberer and *Sanfte Strukturen*, as described by Kalberer & Remann [218,219]. There are many examples of these *Sanfte Strukturen* bundled living willow structures that are of multi-storey height. These examples have only single-storey occupancy however, so they do not test the ability of these structures to support live occupancy loads. Also, the larger of such structures sometimes include metal poles for structural reinforcement, according to [212]. The *Auerstedt Auerworld Palast* by Kalberer & Strukturen^13^ had before 2011 successfully reached mature growth according to the original design and was living healthily for a period of time according to [212], although many of the willows seem to have died and been removed in 2012, according to the website of the project^14^. A similarly large structure, the *Longrun Meadow Willow Cathedral*^15^ shown in figure 6, was constructed in Somerset, UK. The most used of these structures has arguably been the *Rostock Willow Church* by Kalberer & Strukturen^11^ described above for its textile roof, part of the *World Horticultural Exposition* in Rostock, Germany.

#### 3.3.3. Joining constrained growths via grafting

Plants that become woody and structurally stiff in late growth phases can be constrained while they are young, until the plant has matured enough that constraints are no longer needed to keep the plant in position. This strategy can additionally be used with plants that have substantially more structural potential than willow, but of course, these species also have a longer growth period to reach maturity. This method typically incorporates horticultural grafting [220], induced during the process of mechanical constraint. After initiation, constraints apply enough pressure that stems are joined together through growth processes over time. Examples of such structures have been reviewed in part by Ludwig [211], Gale [212] and Katola & Goy [221] in their respective literature reviews. When used to construct sculpture, furniture, and other smaller elements, this strategy is often termed *arborsculpture* or *tree shaping*, and has been used to make a wide variety of growths [211,212,221–225]. Besides trunks or stems, it is also possible to keep partial root systems above ground and shape them, as described for ficus trees by Golan [226].

**Figure 7.**
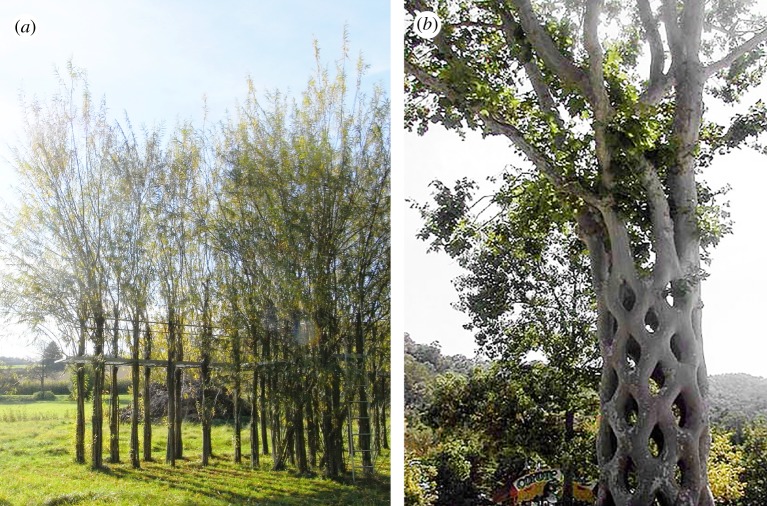
Example methods of combining constrained plant growth with mechanical scaffolds and with grafting. (*a*) An example growth phase of the *Baubotanik Footbridge* by Ludwig *et al.*,^21^ where living trees support a steel platform; image used with license. (Image copyright: F. Ludwig. Image provided by Ferdinand Ludwig, of the *Baubotanik Footbridge* project consortium,^21^ and used with permission.) (*b*) The *Gilroy Gardens Basket Tree*,^16^ where several trees were woven together manually and grafted over time; image used with license. (Image retrieved from Wikimedia Commons, from username Palnatoke. Used with Creative Commons license CC BY 3.0. Image copyright holder chose and approved the license at upload. Image adapted, as permitted by license.) (Online version in colour.)

Several large sized grafted tree sculptures were constructed by Axel Erlandson decades ago [224], and have thus had time to mature. His *Gilroy Gardens Basket Tree*^16^ shown in figure 7*b*, which comprises several trees woven together to form a hollow diagrid-surface column, provides evidence that mature shaped and grafted trees could have structural success at multi-storey heights. Many grafted living structures meant to function as buildings or architectural elements have been begun by Kirsch [225], who according to [212] and [211] has based his process on the historic patents of [227,228]. The *Kassel Waldgartendorf* by Kirsch & Block^17^ showed some success in its middle growth phases, documented by Ludwig [211]. The existing living tree structure that is designed to be functionally closest to an occupied building is the *Ash Tree House* by Kirsch,^18^ planned to have a fully enclosed living roof, fully enclosed living walls with windows, and several subdivided rooms [225]. During its middle growth phases, the *Ash Tree House* also had preparations added for electrical utilities, according to [212]. Its design comprises tightly woven trees with only small gaps between them, meant to eventually graft together into solid continuous walls. This solid living wall strategy however challenges plant health, and according to [211] could not succeed in later phases. A very recently planted structure, *The Patient Gardener* by Visiondivision & di Milano^19,20^ plans to apply the *arborsculpture* approach to construct a two-storey building structurally fit for occupancy. Its design uses living trees as both wall supports and floor supports by planning to bend and join the trees through grafting at mid-height, forming an overall hourglass shape for the structure. Its growth phases are still too early to provide evidence for whether its strategy of acute bending will provide sufficient joint pressure for successful grafting, a primary concern among *arborsculpturists* according to [212].

#### 3.3.4. Combining constrained growth with mechanical scaffolds

In contrast to the fully living structures described above, the literature also includes hybrid approaches, in which constrained living plants are combined with mechanical scaffolds. Two strategies for these hybrid approaches are documented in the literature, one which uses the mechanical scaffold as a temporary mould, and one which embeds the mechanical scaffold into plant tissue and incorporates it permanently as part of the structure.

For the method of using mechanical scaffolds as removable moulds, the examples in the literature are the size of furniture or building components, and plan for the grown object to be harvested at a certain stage, for processing into industrial products. Before the stage of harvesting, [229] strap bamboo onto mechanical profile forms during growth, constraining them in the shape of a vehicle frame. This example is not yet extended to the processing stage after growth. Finished furniture products such as stools, using young trees strapped to small moulds during growth, have been made by Chris Cattle for decades, as described by Johnson [230]. Products such as chairs and lamps are made by Munro & Full Grown [231], using young trees strapped to reusable industrial moulds in a process that nears mass manufacture [232]. An extension of the mould method is investigated by Beger *et al.* [233], using shaped tubes to direct growth, instead of constraining it fully. Though the existing uses of moulds are for furniture-sized elements, and for products that are harvested rather than maintained indefinitely in a living state, similar moulds could be investigated for larger and longer-term growth, with moulds applied incrementally or holistically.

The method of embedding mechanical scaffold in plant stems over time, and thereby creating a biohybrid structural system, has been investigated for the application of multi-storey buildings. The *Baubotanik Footbridge* by Ludwig *et al.*^21^ uses trees as living columns to support a steel platform and handrail at second-storey height, as shown in figure 7*a*. The mechanical platform and handrails maintained their location and orientation throughout growth, as the stems only grew radially in the zone where the mechanical elements were incorporated, according to [234]. Though there were originally trees planted diagonally as well as vertically, the diagonals did not maintain health and did not survive early growth phases. The vertical trees were still healthy 60 years after construction, as documented by Ludwig [234], and had by that time fully encircled the steel railings at their attachment points, embedding the railings into the living trunks. In order to extend these results to taller multi-storey buildings, the *Baubotanik Plane Tree Cube Nagold* and *Baubotanik Tower*, referred to above in §3.1.3, were built by Ludwig *et al.*^3,4^ In these two, free-standing steel structures were first built with columns and floor plates, with the intention to grow trees in a structural frame pattern around permanent floor plate perimeters at each level, until the trees mature enough that they can structurally support the floor plates and the temporary steel columns can be removed [234]. The growth on both of these structures is still too young to provide evidence for multi-level structural frames from living trees.

#### 3.3.5. Shaping plants by robotic control of stimuli

There are some examples of using robotics to steer the shape of plant growth, at a size smaller than a room. These systems trigger behaviours in the plants such as phototropism, by providing stimuli such as a specific spectrum of light. The behaviours of plants that can be interfaced for robotically steered control are reviewed in §2. Centralized robotic control of plant stimuli is explored by Wahby *et al.* [109,162], Hofstadler *et al.* [110] and Wahby *et al.* [163], using a purpose-specific model of plant growth combined with controllers evolved in simulation to predictably steer growth to two-dimensional geometric targets. In this set-up, the plant has no mechanical scaffold, but the height to which it can support itself is not tall enough for building-sized growth. Steering with such stimuli is extended to distributed robotic control and larger sized growth [134,135]. In this set-up, the plants grow along a mechanical scaffold wall and the shape of their growth pattern is guided by stimuli.

### 3.4. Forming building components from amorphous living material

Organisms that produce material or grow to fill available space on a surface or substrate can be used to form or strengthen functional building components. Approaches in the literature include bacterially produced cellulose, growth of mycelium, and bacterially induced cementation.

#### 3.4.1. Cellulose shaped into membranes

Biologically produced cellulose can be shaped into non-load-bearing membranes that can serve as building shading devices, moisture barriers, or air flow barriers. For instance, cellulose produced by bacteria is used by Araya *et al.* [235] to create thin translucent membranes that are not load-bearing but with further development could be used in buildings to mediate the occupied environment (e.g. daylight or wind) and can potentially be self-healing. In the *Gen2Seat* project by Terreform ONE *et al.*^22,23^ bacterial cellulose is used to grow a thin membrane in its final intended position, covering a furniture volume [236]. This approach is envisioned by Terreform ONE *et al.*^24^ to be extended to a building-sized membrane in the art installation *In Vitro Meat Habitat*, by use of cellulose or of laboratory-grown cells from animals [236]. This vision of bacterially produced cellulose formed directly on a building structure could be investigated for development.

#### 3.4.2. Load-bearing mycelium elements

The growth of fungal mycelium (i.e. mushroom roots) into load-bearing building components, sometimes termed *mycotecture*, is seen in several examples in the literature. Mycelium is grown in rectangular substrate-filled moulds to form simple bricks, dried when growth is mature, and used to construct a small vault structure in the installation *Mycotectural Alpha* by Ross & Far West Fungi.^25^ The mycelium bricks made for the vault failed under a sharp point load but could withstand substantial forces if the load was well distributed, according to [237]. Through further investigation, a method was patented by Ross [238] for producing a variety of highly standardized mycelium bricks structurally reinforced by wood or steel. In both reinforced and unreinforced cases, mycelium used in a building envelope can perform thermal insulation functions [237]. Though these investigations are small in size, a publicly occupied mycelium structure of building size also exists in the literature. The partially enclosed *Hy-Fi* building by The Living *et al.*^26^ is single-storey occupancy of multi-storey height and is constructed of unreinforced mycelium bricks joined with fixed connections. Through a combination of finite-element analysis (FEA) and load-testing bricks with different combinations of properties (e.g. grow time, substrate, and fungi nutrients), the bricks were developed to successfully carry their compression and wind loads for that building design and site [239,240]. In the above examples, the mycelium bricks are baked before construction, to stop the growth process. Mycelium building components meant to remain live after construction, to allow new growth to form, are investigated by Mayoral [241] in more intricate strut-and-node shapes. These prototyped live components are not yet tested for their structural performance, compared to the baked mycelium components above. Live unreinforced mycelium bricks are used to construct a small wall in the installation *Mycelium Mockup* by AFJD Studio.^27^ The wall test results are successful in continued growth after construction, by which new mycelium growth bonds neighbouring bricks together and mushrooms grow from the side of the wall, according to [242]. After the exhibition, the wall is dismantled and moved to an outdoor site [242] where the mycelium is intended to contribute to soil bioremediation (i.e. neutralization of contaminants, see [243]).

#### 3.4.3. Microorganisms and biocementation

**Figure 8.**
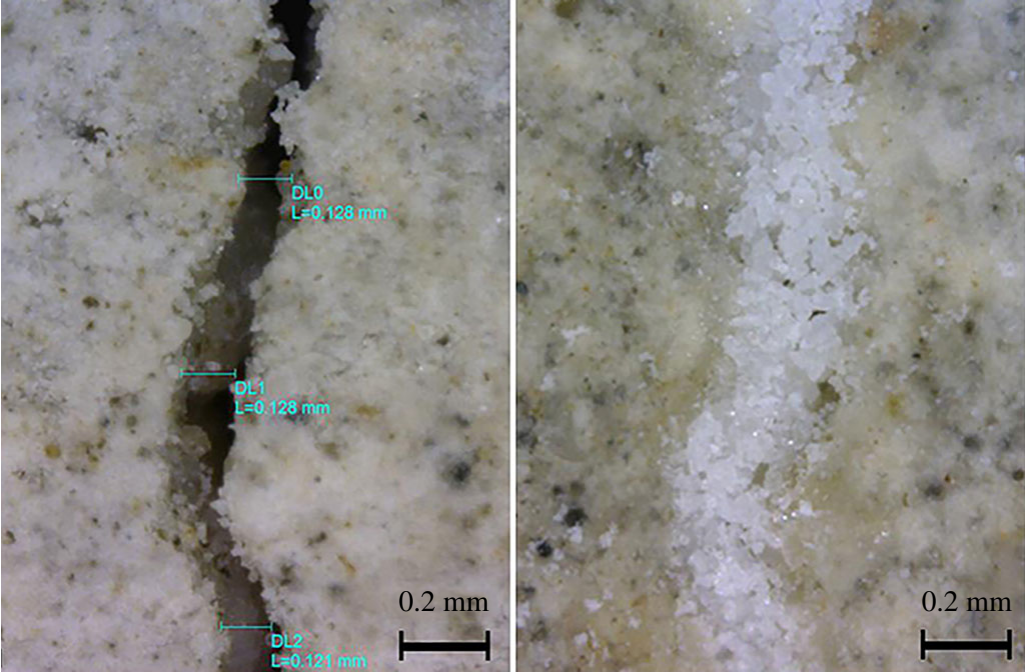
Microbially induced deposition of calcium carbonate for self-healing of cracks in concrete [246], an example of biocementation. Images from [246] and used with license. (Images reprinted from fig. 7 (subfigures *a* and *b*) of the Frontiers in Built Environment paper of Farrugia *et al.* [246], DOI, open access. Used with Creative Commons license CC BY 4.0. Authors holding the image copyright approved the license at publishing.) (Online version in colour.)

Biocementation of soil (i.e. hardening) and bioremediation of concrete structures (i.e. restrengthening of degraded concrete) with certain types of bacteria is a well-investigated area of civil engineering, construction technology, and geotechnical applications, as reviewed by Pacheco Torgal *et al.* [244]. In these applications, the bacteria are not specifically shaped, but rather act to fill any voids or porosity that occurs in the material to which they are added. Microbes that induce the production of minerals through biochemical reactions can be used to form a biocemented crust on a volume, a biocemented layer of a specific depth, or an overall biocementation of an entire monolithic structure [244]. In standard concrete structures such as buildings, bacteria can be intermixed to seal new cracks as they form, as in the examples of [245,246], seen in figure 8. Bacteria can also be intermixed in concrete structures in harsh conditions (e.g. submerged in seawater or toxic materials) to support continual remediation and improve the longevity of the structure, as in the example of [247]. Beyond strengthening concrete, bacteria can cement undisturbed soil *in situ* when added to the top of the volume, percolating down throughout [248]. An extension of this method is envisioned and modelled by the *Computational Colloids* project [249], in which bacteria are genetically modified to induce mineral production in reaction to environmental changes in pressure, forming a self-organizing foundation for a building.

### 3.5. Structural modelling of biohybrid buildings

If biohybrid structural systems are to be built for standard occupation, their features will need to be approved by regulatory bodies. Most of the above examples of publicly accessible structures either might be categorized by their authors as art installations, or are built in isolated terrain where governments might not enforce building code regulations. In order to systematically realize buildings for long-term occupancy with biological elements in a structural role, the biological portions will need to be demonstrated as fulfilling structural provisions of relevant local and international building codes (see [250–253]). Models of structural behaviour will be challenging for materials that are living or are biologically deposited directly on site, therefore including some degree of unpredictability. In the process of developing the aforementioned *Baubotanik* structures, experiments were conducted to modify the structural Young’s moduli of stems of the used plant species. In these experiments, a substantial variety in stiffness was achieved by altering environmental conditions during growth [254].

To predict structural performance in living buildings, we find two categorical approaches in the literature to be evidently relevant, one being FEA and the other being various artificial intelligence methods. FEA, which is standard across engineering disciplines [255], is also used in biological sciences for the study of plant biomechanics, among other functions [256]. This application of FEA could be investigated for extension to biological material in buildings, carrying multi-storey and live occupancy loads. FEA was used, in combination with material testing, to confirm the structural behaviour and safety of the aforementioned *Hi-Fy* pavilion’s fungal mycelium brick structure, in a way that was sufficient to be accepted for temporary public occupancy [239,240]. Further pursuing this approach with the goal to establish biological building blocks in construction, we propose that biohybrid organisms be comprehensively specified in terms of expected environmental conditions in relation to structural and other properties such as amount of bio-material produced or shadow cast. The resulting database could be fed into a general, centrally maintained registry, similar to the one set-up for amino acid chains and proteins for synthetic biology by MIT’s international competition on genetically engineered machines (iGEM). One step further, also considering robustness that can result from sets of biohybrid agents working together, biohybrid (sub-)systems could be specified accordingly. The robots, which can be well-specified to begin with, could also fulfil the task of measuring the plants’ proper development in accordance with the provided registry information and communicate their findings like sensor networks throughout the system and to the human user, in case interference is required.

Though the mycelium in the example above was killed before the bricks were aggregated, the unknowns of the material still caused substantial variation in material performance during the building’s short lifespan. After heavy rainfall, moisture affected the stiffness of the mycelium bricks in a way unanticipated by the engineers, causing large deformations, according to [240]. The most affected areas of the structure were rebuilt during the lifespan of the building, successfully enabling continued public occupancy.

For the second approach, of various artificial intelligence methods, there are examples in the literature used to predict the behaviour of materials that are non-uniform or present other challenges (cf. neural networks for concrete or 3D prints [257,258]; genetic programming for limestone or geopolymers [259,260]. Such methods could be investigated for predicting the structural performance of biological material that is alive or is deposited *in situ*. The modelling techniques used in the context of self-organizing systems (see §§ 4.2 and 4.3.3) could possibly also be applied here; but we are not aware of any related work.

## 4. Robots for biohybrid construction

### 4.1. Centrally controlled robots in construction

Industrial robots have been extensively explored for off-site prefabrication in AEC [261], in ways that have fundamentally shifted AEC design and execution [262–265]. On-site construction automation with industrial robots also enjoys substantial exploration in the literature [266,267]. This realm presents new challenges when compared to prefabrication, as work takes place in unstructured environments rather than laboratory or factory conditions [261]. Improved approaches to existing construction processes are, of course, an important challenge for on-site AEC automation [261]. Perhaps more ambitiously, as noted in an editorial on construction robots by Yang [6], on-site AEC robotic processes may present entirely new types of construction opportunities. In the context of a new type of construction for biohybrid buildings, where biological elements either grow or deposit material *in situ*, we have to take into account uncertainty in terms of sensory information (measurement precision and noise), dynamics in terms of ever-changing environments over different timescales, and diversity in terms of the tasks robots need to fulfil—from planting seeds and watering to self-assembling into scaffolds at high altitudes. The most versatile robot is not a single entity but a collective of robots that self-organize and coordinate their work to achieve goals no individual would by itself. Hardware and software to achieve construction automation via robot collectives is quickly developing [268,269].

### 4.2. Realizing constructive robot collectives

Technically speaking, self-organization can be understood as the distribution of control of a system over a considerable set of its components [270]. This immediately applies to systems comprised of autonomously acting agents, as each of those follows its own agenda. Thus, biological systems are inherently self-organizing. When designing technological systems, one also has to consider that large systems that have to work flexibly and be robust to local failures and changes in the environment, can only be realized if individual components may act autonomously—otherwise, the managerial overhead, the communication overhead and the risk of single points of failure do not allow scaling up of the number of involved components or subsystems [271].

**Figure 9.**
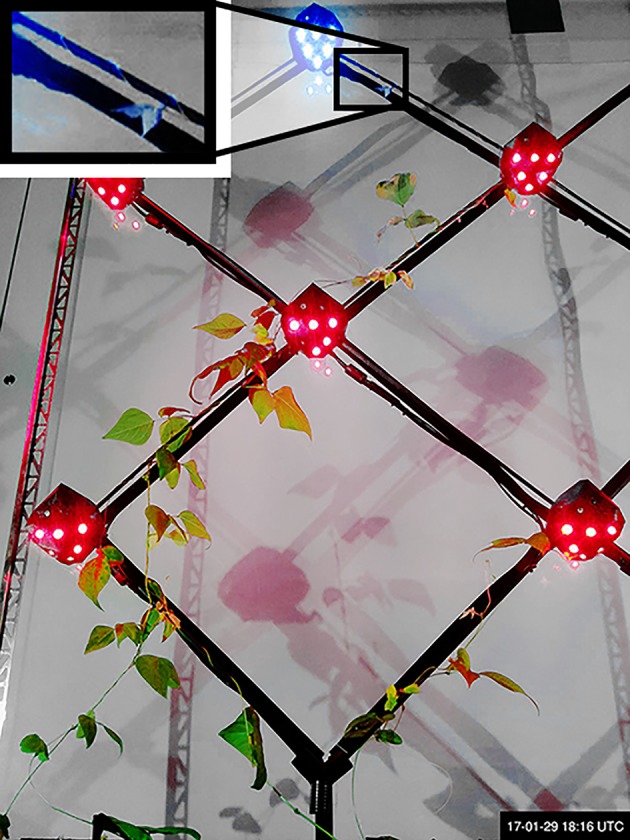
A group of distributed robots providing directional stimuli to steer plant growth on a mechanical scaffold [134]; image from [134] and used with license. (Image reprinted from fig. 12(b) of the Royal Society Open Science paper of Wahby *et al.* [134], DOI, open access. Used with Creative Commons license CC BY 4.0. Authors holding the image copyright approved the license at publishing.) (Online version in colour.)

In the context of biohybrid systems, where large numbers of agents or robots might be deployed to interact with plants or animals in various ways, the capability to also concert robotic construction efforts (e.g. to provide scaffolding for the plants’ growth) is crucial. The intelligence of such robots has to consider their environment and to closely align their activity with their biological counterparts. The ability to quickly adapt to new situations, for instance, if a plant branches out, without losing the user-defined goals out of sight, for instance to grow in height, requires the robots’ controllers to handle a great variety of situations. Even in cases where the possible growth directions are intentionally restricted, as seen in figure 9, it has been shown that the task of robotically managing several plants simultaneously is quite complex [134,135]. The variety of goals, the expected flexibility, the complexity of the interactions in biohybrid systems, and in addition, the uncertainties and insufficient precision in perceiving and manipulating real-world environments would require the robots to learn [272]. If we can narrow down the tasks of a specific robotic unit, we may be able to find a simple, reactive behaviour that renders collaborative work possible [10] and robustly succeeds even within a broad range of situations [270]. Yet, even the realization of a modestly simple robotic unit that could grow artefacts and thereby guide and support biohybrid development is already challenging as stressed in the following paragraphs.

#### 4.2.1. Materials for self-organized construction

In order to conceive both robotics hardware and self-organized behaviours for construction tasks in the context of biohybrid systems, we first shed some light on rigid and amorphous materials—the two categories that have been considered in the literature.

Magnenat *et al.* [273] made robots deploy cubic bricks to bridge gaps and to stack them up as tower constructions. Consistent alignment and cohesion between the bricks was established by magnets. Similarly, [37] made use of deployment-ready building blocks. In order to better support structural loads, protrusions on the surface ensured a tight bonding mechanism between the elements. Aluminium rods were deployed by Stroupe *et al.* [274]. Its size rendered collaborative transportation by two robots necessary. An alternative, also to render the transport easier is realized by blocks of polyurethane foam [275]. The foam blocks were glued together applying an adhesive. A less persistent approach is to establish magnet bonds by means of electronic components as realized by Werfel *et al.* [276,277].

Napp & Nagpal [278] used amorphous foam to construct ramps to elevate grounded robots to higher construction levels. In order to compensate for uneven surfaces, the flexibility of amorphous materials was harnessed. Napp & Nagpal [279] later succeeded in constructing larger volumes using these ramps and foam material. Previously, [280] had tested toothpicks (with glue on their tips), sandbags (with rice and corn to fill the gaps) similar to [281], and said foam. The resulting artefacts were examined for features such as sensitivity to pressure, effort of transportation and deployment and associated costs. Depending on the context, different materials are favourable. The expansion of foam, for instance, facilitates storage and transport but incurs greater costs. Sandbags are cheaper and the resulting construction is immediately usable, which is important in self-organizing systems as otherwise the robots need to synchronize their construction efforts. In order to achieve greater versatility, [282] mixed two-component polyurethane and right away printed the material by an airborne robot. In the context of airborne construction, there have also been efforts to let quadcopters build tensile structures from threads or ropes [283–285].

#### 4.2.2. Robotic hardware for self-organized construction

Considering hardware options for realizing self-organizing robotic communities for the purpose of construction, there are mainly the two categories of ground and airborne units.

Most ground robots follow an approach that is also represented by the marXbot by Magnenat *et al.* [273] and Soleymani *et al.* [286] or the Swarm Robotics Construction System (SRoCS) by Allwright *et al.* [287]. The marXbot’s small and lightweight base is augmented with a basic set of sensors including a rotating distance sensor, 24 ultrasonic sensors and eight ground sensors. Its battery lasts for up to 7 h. As actuators, the marXbot is equipped with two magnetic arms, whereas the SRoCS realizes grabbing by means of a fork-lift. Working with the marXbot and alike can be challenging. For instance, although they are augmented with magnets, its grabbers may not work as expected for transporting and deploying construction elements as emphasized by Karakerezis *et al.* [288]. Another challenge lies in the need to recharge the battery; it could tap into environmental resources such as solar power and recover during a long break or to visit an energy outlet, which requires complex planning and path-finding routines. Directing the robots across a dynamic construction site can be a demanding chore in itself. Nigl *et al.* [289], for instance, guide construction robots by means of rails. There are also conceptual works such as by Saltarén *et al.* [290] which shed light on the robots’ movement capability in more complex scenarios, for instance if the robot needs to climb the built structure to manipulate it. In the long run, robots might become capable of reconfiguring themselves, thus changing their shapes and functionalities as outlined by Rus *et al.* [291]. Clearly, such concepts bear numerous additional challenges but they might also hold the key to versatile robotic systems needed to not only build by themselves but also to actively support and direct plant growth in biohybrid systems.

For the immediate realization of biohybrid systems, either ground or airborne units can be chosen. Flight opens an additional spatial, navigational dimension compared to grounded units. But flight also means that minute errors may quickly lead to crashes that result in complete failures and loss of hardware. Precautions must be taken accordingly—for instance by provision of accurate values of remaining energy. Due to their reliable and robust flight, quadcopters have been studied in the context of construction tasks [275,282–285,292,293]. In airborne contexts, however, the transport and deployment of construction materials is even harder than on the ground. A systematic inquiry on handling construction materials in airborne set-ups was conducted by Mellinger *et al.* [292]. It revealed the crucial role of the relative position of the construction material both for transport and deployment.

#### 4.2.3. Discussing options for deployment

The precision and supposed ease of deployment of rigid construction materials greatly depends on the rigour of the building blocks’ manufacturing process. In addition to these efforts, there are other drawbacks such as the need for pre-designed joint mechanisms or the use of additional adhesive materials, as well as an inability to build directly on uneven terrain. However, rigid materials can bring about great stability. Obviously, the less precise but adaptive and *ad hoc* deployable amorphous materials can compensate for the lack of flexibility of rigid materials. Therefore, [268] concluded that a multi-stage process that considers different materials at different times, similar to traditional building construction, might be most beneficial. They also suggested that a heterogeneous set of airborne and ground robots might be most successful considering their individual strengths and weaknesses—high risks but easy manoeuvrability of airborne units and inflexible but strong and robust grounded robots.

### 4.3. Control, collaboration and modelling

In general, the control of collective robot systems is challenging. The usual approach is to keep the individual, local controllers simple and create complexity from interactions between robots. While system complexity can also be kept low by letting the robots work in parallel without explicit robot–robot interactions, the more ambitious objective should be to let them closely interact and to create true collaboration between the constructing robots beyond mere parallelization. The robot controller design can be supported by models for better predictions about the expected global behaviour.

#### 4.3.1. Control

Construction of living buildings by biohybrid robots is currently too underexplored for the literature to include established, purpose-specific approaches to control. Instead externally standard approaches are used and novel approaches are borrowed from other fields. Here, we restrict our discussion mostly to multi-robot systems. The standard approach in multi-robot set-ups is to limit the robot controllers to simple behaviours for two reasons. First, multiple interactions between robots complicate the system [294,295], hence, one wants to keep as many components simple and manageable as possible. Second, the idea is to create complex behaviours from the interactions between robots and their collaboration, not from complex individual behaviours. This is in line with the concepts of swarm intelligence [296] and emergence [297].

The applied underlying concept for these rather simple controllers is often behaviour-based robotics, such as the subsumption architecture by Brooks [298]. The approach by Mellinger *et al.* [299] uses standard techniques of (centralized) control theory. Allwright *et al.* [287] use an *ad hoc* approach resembling partially the idea of behaviour-based robotics. Werfel *et al.* [37] use reactive control based on behavioural rules. The main research question here is, how to derive or generate these rules (see §4.3.3).

#### 4.3.2. Collaboration

**Figure 10.**
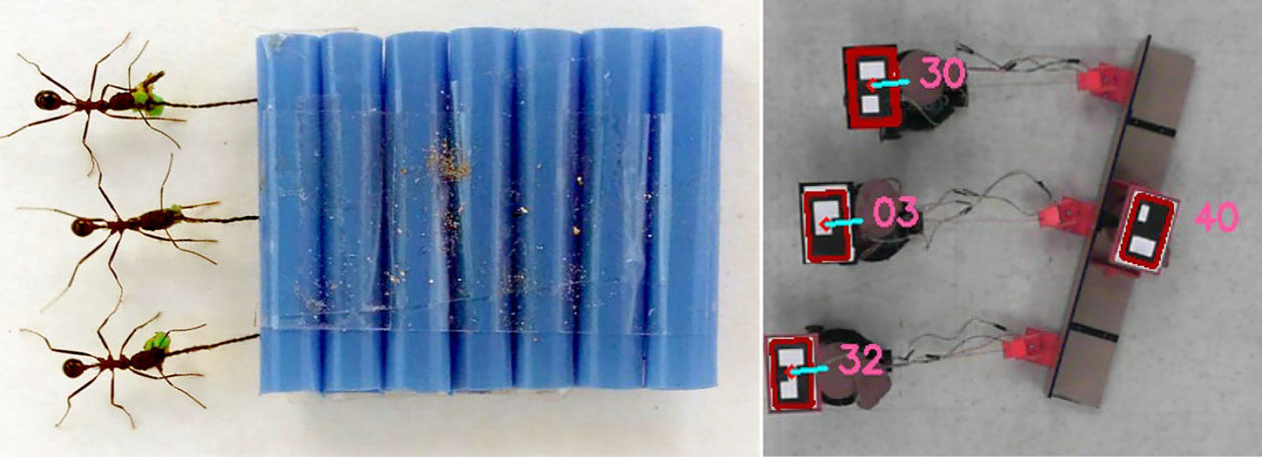
Collaboration of multiple robots on the construction sub-task of towing materials, inspired by a collaborative mechanism in social insects [300]; image from [300] and used with license. (Image reprinted from fig. 1 (subfigures *a* and *b*) of the Royal Society Open Science paper of Wilson et al. [300], DOI, open access. Used with Creative Commons license CC BY 4.0. Authors holding the image copyright approved the license at publishing.) (Online version in colour.)

In multi-robot set-ups, the questions arise of whether and how the robots should collaborate. Often the robots work in parallel but rather independently (see for example collaborative material towing in [300], shown in figure 10). An immediate challenge in multi-robot scenarios is that robots have to avoid collisions between each other. In addition, each robot should be granted access to shared resources (e.g. space, charging stations, etc.), both deadlocks and bigger interference effects should be avoided, too [301]. However, the ambition should be to go beyond a mere concurrent parallelization and enable the robots to collaborate. Then one can hope for super-linear performance increases [302–304] and for self-organization into higher order entities, i.e. teams, taking care of different parts of the task [305,306]. Efficient collaboration between robots requires robot–robot communication. An option is to use direct point-to-point communication, however, often it is advantageous to allow for asynchronous communication. Construction usually requires that robots place building material at well-defined positions, sometimes coordination between robots may be required, and robots may not always meet at the material destination site to directly communicate the position of the building material to be added next. Following again the concepts of swarm intelligence, an option is to use *stigmergy* [25], that is, asynchronous communication via the environment (see §2.1.1). Stigmergy in construction usually means that the presence or absence of building material itself is used as cue [296,307]. The robots then have simple rules when to place material where depending on the current, local state of construction (cf. the wasp nest construction model by Theraulaz & Bonabeau [34] discussed in §2.1.1). The designer of the system has to take care that the summation over all these simple behaviours results in the desired construction without deadlocks (e.g. certain areas cannot be reached anymore after placement of building material in unanticipated sequences). This approach, however, still has a tendency to mere parallelization. True collaboration would arise once robots hand over building material, collectively transport bigger pieces, and maybe even self-assemble, for example, to reach high positions.

#### 4.3.3. Modelling

As mentioned above, controlling interacting robots is already a challenge but the control of multi-robot construction even more so. If the robot controllers follow the concept of self-organization with a strict limitation to local information to stay scalable, then the overall system is difficult to govern. Besides the standard tool of simulations [308], in multi-robotics one also uses modelling techniques to predict expected system behaviours. Specific for multi-robot biohybrid systems for construction are the requirements of spatial representation in the models and support for multiple timescales. There are non-spatial models based on rate equations in swarm robotics [301,309] that have successfully been applied to different scenarios. However, for construction it seems essential to represent space, hence, represent intermediate configurations of the construction in space and time. Options are models operating on continuous space [295,310] or discretized space [37]. The discrete case seems a considerably simpler approach, especially if the building material is also discrete (e.g. bricks). Modelling, control and construction are more challenging if the building material is continuous [311]. In order to realize self-organizing buildings for occupancy, it is necessary to satisfy government regulations that are standard for AEC sectors (e.g. [250]), as described in §3.5, meaning that details of the final structure must be somehow guaranteed before construction begins. Werfel *et al.* [37] address this by providing each mobile robot with the plan for the final structure. Architects suggest another approach whereby approval of a fully detailed plan might not be necessary as long as the key features of the structure can be guaranteed [312].

Support for multiple timescales is important once mobile robots and/or human beings are combined with either natural plants or material-depositing animals. Timescales relevant for mobile robotics and humans are seconds or fractions of seconds, while relevant timescales for growth and motion of natural plants and animals’ nests are hours, days, or even weeks. Modelling techniques would hence be necessary to generalize from small time-step phases to big time-step phases (roughly relating to the technique of adaptive stepsize in numerical analysis).

### 4.4. Human–biohybrid interfaces

Interaction with machines has been a challenge ever since machines came about. The research discipline of human–robot interaction (HRI) especially focuses on automata that can behave autonomously and their interactions with humans. A comprehensive introduction is provided by Goodrich & Schultz [313]. HRI aims at discovering new insights about interfaces for various degrees of autonomy—from direct teleoperation of a robot to its full autonomy—and for various situations involving one or more robots as well as humans. In HRI settings, robots generally assume one of the following roles: supervisor, operator, mechanic, peer, bystander, and mentor. In the context of biohybrid systems, all these roles make sense but their objective also extends beyond the human user to the other system components. For instance, they can assume roles in relation to the other robots, which is addressed by the research areas of multi-agent [314], self-organizing [270], and complex systems [315] as well as, specifically, by swarm robotics research [294,316]. We focus below on the particular challenge of HRI interfaces for a human user that needs to guide an otherwise self-organizing biohybrid system. In addition to guiding the system, according interfaces also need to provide information about the current state of the system and its potential development.

#### 4.4.1. Biohybrid design and control

**Figure 11.**
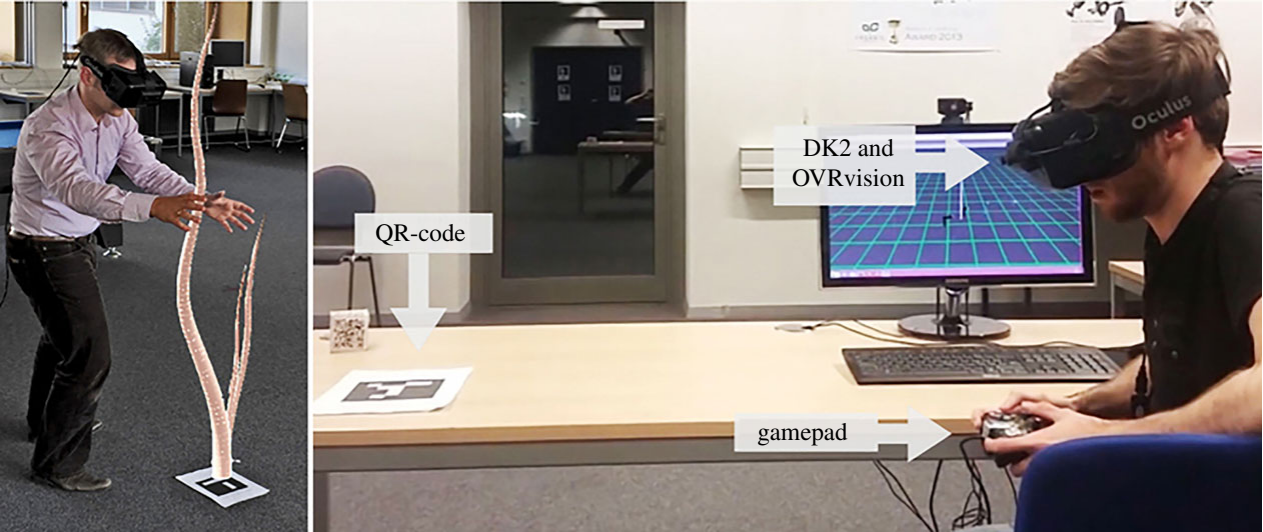
Augmented reality interfaces for user interaction with simulations of biohybrid living construction [318]; image from [318] and used with license. (Images reprinted from fig. 8 (subfigures a and b) of the Frontiers in Robotics and AI paper of von Mammen *et al.* [318], DOI, open access. Used with Creative Commons license CC BY 4.0. Authors holding the image copyright approved the license at publishing.) (Online version in colour.)

von Mammen *et al.* [317,318] presented a prototype of an augmented reality interface for biohybrid system design (figure 11). They outfitted the user with a head-mounted display augmented with a pair of cameras to provide a stereoscopic video feed of the environment. This video feed could be overlaid with information about a simulated biohybrid system. In the given case, the user was able to seed simple plant-like structures that would grow upwards and towards light sources. The strategic placement of lamps allowed the user to steer the structural growth, for instance, to climb around a pole. This augmented reality (AR) prototype already hints at the potential design and use-case for the next generation of AR prototypes for biohybrid system design and control. In addition to the different kinds of system components that could be deployed (plants and lamp-‘bots’) and configured (at least the technical devices), the system allowed the user to fast forward into the near future and explore the result in a real-world context. Heinrich*et al.* [319] explored user control of self-organizing construction more generally, through an interactive evolution approach.

#### 4.4.2. Guiding biohybrid swarms

Human–swarm interaction (HSI) can be considered a subset of HRI research with a focus on controlling and inspecting collective robotic systems (e.g. [320,321]). A rather recent review on HSI is provided by Kolling *et al.* [322]. As pointed out by Bashyal & Venayagamoorthy [323], in HSI questions of scalability, harnessing the system’s intelligence and working with locally available knowledge are of special interest. Due to the complexity that can arise in HSI scenarios and that systems comprised of large numbers of interacting components lend themselves well for distributing activities, use-cases with multiple users are frequently considered as well (e.g. [324–326]). Again, a wide spectrum from direct control to full autonomy of the swarm is considered, with intermediary steps being realized by either hierarchies in command unfolding across the systems’ constituents or by means of more or less abstract goal formulations by the users.

#### 4.4.3. Conclusion on human–biohybrid interfaces

Research towards interfaces between humans and biohybrid systems is at an early stage. The target domain of biohybrid systems yields new challenges or intensifies those considered by HRI and HSI. For instance, different from interactive with robot collectives only, there is the need to model behaviours of reactivity and development of the inherently heterogeneous population of organisms in varying environments. This directly impacts the responsiveness to various user-induced stimuli and necessitates thinking in probabilities or ranges of outcomes. The timescales involved pose another challenge that needs to be addressed. The individual lifespans of the organisms, their developmental stages, the interaction with the environment—all these aspects may play out on different dimensions of time. This insight also reinforces the important role that simulations will play for the informed design of biohybrid systems.

## 5. Discussion

Living organisms as building components have to be considered not only as continually growing entities, but also as dynamic, open systems that change structurally and morphologically in time. Many species are subjected to regular changes. For instance in plant organs, mechanical properties change due to the seasons and the developmental stages, and annual plants do not disappear after dying but will continue to mechanically impact the system. In a living system, certain animal depositions and plant organs not only develop but may spontaneously be withdrawn if they are no longer fulfilling their intended role. Planning and coordination of biohybrid construction processes will involve cycles of spatial expansion and reduction.

Living organisms sense and respond to environmental changes by adjusting their internal processes to overcome threats and to take advantage of changed conditions. Organisms successfully realize their developmental programmes due to their plasticity. In addition, organisms actively shape their environment. For example, trees change light conditions for their lower branches, they change the soil structure, underground water conditions, and the ambient air. The activities of living plants change the originally provided conditions, such that future growth is not guaranteed. In biohybrid construction, environmental conditions and the physiological reactions of organisms will have to be monitored and perhaps modulated continually, on long timescales and large spatial scales.

The artificial elements of a biohybrid system also influence the environment. There are intentional influences, by stimulating physiological reactions or providing scaffolds, but there can also be side effects. For example, robots will increase the temperature locally due to waste heat, influencing animal behaviours and plant generative organs in close proximity. This may not be harmful; flowers generate complex heat patterns to attract and assist pollinators towards flowers. In biohybrid construction the system will need to autonomously deal with non-anticipated situations—a pervasive challenge throughout robotics, which is not yet solved. One advantage of approaching this challenge within a biohybrid system is that many actions may be required only on intermediate and long timescales, compared to typical robotics applications.

The slow speed of biohybrid construction compared to standard construction may be its primary limitation, especially if the structure is based on woody plant species or other processes that last several decades. In addition, the considerations we have previously raised [327] for biological-engineered hybrids generally are still relevant in the case of application to buildings, and may raise further domain-specific limitations. Future work in the fields of gene modification or synthetic biology may help to ameliorate limitations, either by making growth speeds faster or making grown or deposited materials stronger. Research has advanced plant genetic engineering for instance to improve their performance as biofuel [328]—it may indeed be feasible to improve their performance as living structures for occupancy.

## 6. Conclusion

Here, we have reviewed the existing understandings, technologies, and approaches that have contributed to the development of biohybrid living buildings and construction, or could be used in future studies targeting the relevant challenges. We have reviewed biological organisms and behaviours that deposit, shape, or otherwise generate material in a responsive and typically directional manner. We have also reviewed the methods and technologies that have coupled biological organisms with mechanical elements, integrated them into a construction process or infrastructure outcome, or coupled them with robots. Finally, we have reviewed the autonomous approaches, namely those that are self-organizing, that we expect to be relevant when targeting construction that incorporates both robots and biological organisms. In the abstract and introduction, we note that the targeting of biohybrid living buildings is in part driven by the advantages that living material may offer over traditional synthetic alternatives, and throughout the review, we examine the literature for the occurrence of these advantages. We find that both the self-repair of damage to a living or synthetic structure and the resilience to corrosive environments, achieved via biological organisms, has been demonstrated several times in the literature, prominently in the use of *Ficus elastica* roots in the *Living Root Bridges* [209] and of bacteria in the remediation of concrete [247]. We find that an increase in structural performance over time, as opposed to degradation, has been demonstrated in examples where woody plants form part of a load-bearing structure, notably in the *Baubotanik Footbridge*.^21^ We find that support for ecosystems, soil remediation and biodiversity have often been proposed as key targets and challenges, such as by Mohamed *et al.* [148] to combat desertification with robots, but that examples of successful technological implementations remain a gap in the literature, in the topic of biohybrid living buildings. We find that mitigation of the urban heat island effect is regularly targeted by well-established technologies such as green roofs [191], but that integration of this objective into biohybrid robots or construction processes is a remaining challenge. In conclusion, we find a high number and wide variety of references that handle some combination of living organisms, robots, and buildings and construction. However, we find that these examples are quite disparate from one another, and that the field has broad gaps and remaining challenges to achieve construction of a biohybrid living building.

## Endnotes

1 Urbano E *et al*. 2007 Eco boulevard in Vallecas. Images by the architect and republished in [221], 66. Building in Vallecas, Madrid, Spain.

2 This citation, like several others in this paper, may look odd, as it cites a company as an author. Buildings, like paper figures, are citable works of intellectual property; example of how to cite images refer to [329]; addition of buildings as of 1990 refer to [330]. If the team of architects or builders has not written a scientific paper about their structure, then we cite the third-party author who has published a description and photograph(s) of the original work, from which we were able to understand the structure. In this case, the citation style we have chosen is such that the in-text citation (often including a company as an author) is for the original structure, and its respective bibliography entry in turn points to the third-party source publishing its image.

3 Ludwig F, Hackenbracht C, Baubotanik research group, and Neue Kunst am Ried. 2009 Baubotanical Tower. Images by author and published in [333], 86. Building in Wald-Ruhestetten, Germany.

4 Ludwig F, Schönle D, Brocke I, Roesler C (SecOp/GaLaTech). 2012 Platanenkubus Nagold (Plane Tree Cube Nagold). Images by author and published in [211], 254–255. Also published in [196]. Building at the 2012 Landesgartenschau in Nagold, Germany.

5 Genetic Architectures Research Group, Estévez AT. 2008 Biolamps: Genetic barcelona project, 2nd phase. Images by architect and published in [206], 452. Room interiors at a private building in Barcelona, Spain.

6 See endnote 2

7 PHILIPS. 2010 Microbial home: Biolight. Referenced without images in [207], 6. Product prototype.

8 Burggraf N, Zauner S, and Thierfelder H. 2010 Bioluminescent field. Images by designer and published in [208], 84, 191. Exhibit at Luminale 2010, Frankfurt Lighting Culture Biennale.

9 Gale B. 2011 Living willow tunnel. Published in [212], 18. Built structure at Pierce’s Park in Baltimore, Maryland, USA.

10 Schaeffer J, Kotin S, and Tebbutt C. 1996 Willow dome. Early growth images published by the architects in [331], later growth image by author published in [215], republished in [212], 3. Built structure at the Real Goods Solar Living Center in Hopland, CA, USA.

11 Kalberer M, Strukturen S. 2003 Willow church. Images published in [211], 48, and [332], 23. Building at the 2003 World Horticultural Exposition in Rostock, Germany.

12 See endnote 2

13 Kalberer M, Strukturen S. 1998 Auerworld Palast. Images by author and published in [212], 21, and [332], 20–21. Building in Auerstedt, Germany.

14 http://www.auerworld.com/auerworldpalast/

15 Jennings S, Courtier S, Project Taunton. 2011 Longrun meadow willow cathedral. Building that is a part of Project Taunton, located in Somerset, UK.

16 Erlandson A. 1940s Basket tree. Images by author published in [222], republished in [211], 37, c. Built structures originally at Tree Circus in Scotts Valley, California, USA, currently at Gilroy Gardens in Gilroy, California, USA.

17 Kirsch K, Block HF. 1993-1997 Waldgartendorf. Images by author and published in [211], 50. Built structures near Kassel, Germany.

18 Kirsch K. 1990 Ash tree house or ash tree dome. Images by author and published in [212], 31, ca. Built structure in Bauhaus, Germany.

19 Visiondivision and Politecnico di Milano. 2011 The patient gardener. Images by author and published in [212], 35, Built structure in Milano, Italy.

20 See endnote 2

21 Ludwig F, Storz O. 2005 Baubotanik research group, and Neue Kunst am Ried. Baubotanik footbridge. Images by author and published in [234], 184. Built structure in Wald-Ruhestetten, Germany.

22 Terreform ONE, Genspace, Ecovative Design LLC, Mitchell Joachim, Oliver Medvedik, Melanie Fessel, Maria Aiolova, Ellen Jorgenson, Shruti Grover, James Schwartz, Josue Ledema, Tania Doles, Philip Weller, Greg Pucillo, Shivina Harjani, Jesse Hull, Suzanne Lee, BioCouture, and NYU Gallatin. Gen2seat: Genetic generation seat. Images by author and published in [236], 2012. Furniture installation in New York, NY, USA.

23 See endnote 2

24 Terreform ONE, Genspace, Mitchell Joachim, Eric Tan, Oliver Medvedik, and Maria Aiolova. 2008 In vitro meat habitat. Images by author and published in [236], Art installation at Genspace in New York, NY, USA.

25 Ross P, Far West Fungi. 2009 Mycotectural alpha, a prototype teahouse. Images by author and published in [237]. Temporary installation at the Eating the Universe exhibition at Kunsthalle Düsseldorf in Düsseldorf, Germany.

26 The Living, Ecovative Design, 3M, Arup, Atelier Ten, Build It Green!, Autodesk Dynamo, SCAPE Landscape Architecture, Advanced Metal Coatings Incorporated, and Associated Fabrication. 2014 Hy-fi. Images by architect and published in [239] and [240]. Temporary building at the Museum of Modern Art (MoMA) PS1 in New York, NY, USA.

27 AFJD Studio. 2015 Mycelium mockup. Images by Krista Jahnke and published in [242], 121–124. Temporary installation at the CATALYZE exhibition of the annual conference of the International Society of Electronic Arts, at the Museum of Vancouver in Vancouver, BC, Canada.

## Data accessibility

This article has no additional data.
